# Supplementary material for: Exploring the potential effect of paricalcitol on markers of inflammation in de novo renal transplant recipients
Source: PLoS One. 2020 Dec 16;15(12):e0243759. doi: 10.1371/journal.pone.0243759 (PMC7743930; doi:10.1371/journal.pone.0243759)
Supplement: S1 Table — (DOCX) [file pone.0243759.s001.docx]

| **Biomarker**  **(plasma levels)** | **Paricalcitol (n=33) Control (n=34)** | | | | | | ***t*-test** |
| --- | --- | --- | --- | --- | --- | --- | --- |
|  | **Baseline**  **Mean (SD)** | **1-year**  **Mean (SD)** | **Change (%)** | **Baseline**  **Mean (SD)** | **1 year**  **Mean (SD)** | **Change (%)** | **p-values (CI ng/ml) for group differences in change** |
| Angiopoietin-2 (ng/ml)*  sCD14 (ng/ml)  sCD163 (ng/ml)  DLL1 (ng/ml)  Endostatin (ng/ml)  MMP9 (ng/ml)  sTNFr1(ng/ml)  Galectin-3 (ng/ml)  NGAL (ng/ml)  vWF in % of ref.plasma*  ActivinA (ng/ml)*  OPG (ng/ml)  TIMP-1 (ng/ml) Neopterin (nmol/L)*  **Per-protocol population.** *T*-test for difference in change: p-values presented with corresponding confidence intervals (CI).  DLL1, delta like canonical Notch ligand 1; MMP9, matrix metalloprotease-9; sTNFR1, soluble tumor necrosis factor receptor-1; NGAL, neutrophil gelatinase-associated lipocalin ; vWF, von Willebrand factor; OPG, osteoprotegerin; TIMP-1, Tissue inhibitor of metalloproteinase 1  Data expressed as mean (standard deviation) or * median (interquartile range).  Continuous data expressed as mean (standard deviation) or * median (interquartile range). | 0.76 (0.52)  1.57 (0.22)  445 (236)  8.89 (2.13)  99.2 (22.4)  102.7 (68.3)  1.96 (0.68)  1.44 (0.56)  290 (120)  82.5 (85.9)  322 (773)  0.92 (0.38)  109 (23)  20.7 (19.1) | 0.71 (0.42)  1.58 (0.30)  559 (321)  9.80 (2.91)  94.7 (21.9)  75.2 (44.9)  2.05 (0.92)  1.19 (0.55)  314 (148)  56.7(36.6)  272 (1036)  1.12 (0.44)  111 (27)  21.4 (14.5) | - 6.6  + 0.0  + 25.6  + 10.2  - 4.5  - 26.8  + 4.6  - 17.4  + 8.3  - 31.3  - 15.5  + 21.7  + 1.8  + 3.4 | 0.70 (0.57)  1.58 (0.24)  479 (250)  9.66 (2.14)  106.6 (27.4)  79.6 (36.5)  2.31 (0.76)  1.44 (0.57)  300 (142)  99.8 (119.4)  554 (876)  1.02 (0.42)  125 (30)  22.5 (13.5) | 0.67 (0.58)  1.52 (0.29)  528 (196)  9.87 (2.68)  101.8 (30.8)  80.3 (50.0)  2.20 (0.71)  1.26 (0.44)  308 (130)  61.4 (52.7)  688 (853)  1.08 (0.42)  125 (38)  20.9 (10.8) | + 4.3  - 3.8  + 10.2  + 2.2  - 4.5  + 0.9  - 4.8  - 12.5  + 2.7  - 38.5  + 24.2  + 5.9  + 0.0  + 7.1 | 0.611 (-0.59 – 0.35)  0.412 (-0.09 – 0.22)  0.263 (-49.6 – 179)  0.207 (-0.40 – 1.79)  0.975 (-11.1 – 11.5)  0.076 (-59.5 – 3.05)  0.274 (-0.16 – 0.56)  0.800 (-0.28 – 0.21)  0.630 (-50.3 – 82.4)  0.357(-22.3 – 61.0)  0.785 (-300 – 228)  0.041 (0.01 – 0.28)  0.724 (-10.1 – 14.5)  0.727 (-12.4 – 17.7) |

**S1 Table**
