# Supplementary material for: Exploring the potential effect of paricalcitol on markers of inflammation in de novo renal transplant recipients
Source: PLoS One. 2020 Dec 16;15(12):e0243759. doi: 10.1371/journal.pone.0243759 (PMC7743930; doi:10.1371/journal.pone.0243759)
Supplement: S2 Table — Results for patients with non-inflammatory cause of end-stage kidney disease. (DOCX) [file pone.0243759.s002.docx]

| **Non-inflammatory cause of end-stage kidney disease** | **Paricalcitol (n=22) Control (n=24)** | | | | | | ***t*-test** |
| --- | --- | --- | --- | --- | --- | --- | --- |
| **Biomarker**  **(plasma levels)** | **Baseline**  **Mean (SD)** | **1-year**  **Mean (SD)** | **Change (%)** | **Baseline**  **Mean (SD)** | **1 year**  **Mean (SD)** | **Change (%)** | **p-values (CI ng/ml) for group differences in change** |
| MMP9 (ng/ml)  OPG (ng/ml) | 103.4 (72.9)  0.98 (0.25) | 63.1 (36.2)  1.23 (0.37) | -39.0  +25.5 | 75.5 (34.4)  1.16 (0.54) | 81.0 (44.8)  1.25 (0.58) | +7.3  +7.8 | 0.018 (-83.3 – -8.4)  0.054 (0.00 – 0.32) |
| **No rejection during study period** | **Paricalcitol (n=33) Control (n=34)** | | | | | | ***t*-test** |
| **Biomarker**  **(plasma levels)** | **Baseline**  **Mean (SD)** | **1-year**  **Mean (SD)** | **Change (%)** | **Baseline**  **Mean (SD)** | **1 year**  **Mean (SD)** | **Change (%)** | **p-values (CI ng/ml) for group differences in change** |
| MMP9 (ng/ml)  OPG (ng/ml)  Intention-to-treat population. *T*-test for difference in change: p-values presented with corresponding confidence intervals (CI).  MMP9, matrix metalloprotease-9; OPG, osteoprotegerin  Continuous data expressed as mean (standard deviation) or * median (interquartile range). | 101.4 (69.1)  0.93 (0.36) | 75.6 (44.6)  1.13 (0.43) | -25.4  +21.5 | 77.0 (37.6)  1.04 (0.49) | 76.1 (40.9)  1.12 (0.54) | +1.2  +7.7 | 0.086 (-53.3 – 3.6)  0.048 (0.00 – 0.24) |

**S2 Table**
