# Supplementary material for: Exploring the potential effect of paricalcitol on markers of inflammation in de novo renal transplant recipients
Source: PLoS One. 2020 Dec 16;15(12):e0243759. doi: 10.1371/journal.pone.0243759 (PMC7743930; doi:10.1371/journal.pone.0243759)
Supplement: S1 File — (DOC) [file pone.0243759.s003.doc]

# INTRODUCTION

The introduction of the immunosuppressive agent cyclosporine A (CsA) significantly improved the outcome of clinical renal transplantation in the eighties by reducing acute rejection rates and increasing short-term patient and graft survival. CsA and the later introduced drug tacrolimus**,** both known as calcineurin inhibitors (CNIs), are presently the cornerstones of the immunosuppressive protocols. Although CNIs have a well documented prophylactic effect against acute rejections, they contribute to the development of chronic allograft nephropathy (CAN)/interstitial fibrosis and tubular atrophy (IF/TA) leading to a substantial number of graft loss, up to 50%, in the long term (Nankivell et al., 2003)

Chronic CNI nephrotoxicity is characterized by arteriolar hyalinosis, striped interstitial fibrosis (IF) and tubular atrophy (TA), and even low doses of CNIs carry a risk of cumulative nephrotoxicity (Myers et al., 1984; Chapman et al., 2005). In order to reduce such allograft damage, current practice is to lower CNI exposure using therapeutic drug monitoring. However, complete CNI avoidance early post-transplant is associated with an unacceptably high incidence of acute rejection (Asberg et al., 2006). The mechanisms for CsA-induced nephropathy are still incompletely understood and the molecular basis remains undefined. CsA leads to activation the intrarenal renin-angiotensin system, which reduces renal blood blow through the action of angiotensin II. Angiotensin has proinflammatory and fibrogenic effects mediated by transforming growth factor beta (TGF-β) signalling. CsA also increases apoptosis in tubular and interstitial cells (Naesens et al., 2009). CNIs may also directly upregulate TGF-β in tubular epithelial cells (Vieira, Jr. et al., 1999; Johnson et al., 1999). TGF signalling may initiate epithelial-to-mesenchymal transition (EMT) leading to tubular degeneration and tubular atrophy (Hazzan et al., 2011).

Paricalcitol (19-nor-1,25-dihydroxyvitamin D2) is a synthetic, selective 3^rd^ generation vitamin D receptor activator (VDRA) indicated for the prevention and treatment of secondary hyperparathyroidism associated with chronic kidney disease stage 3, 4, and 5 (Cozzolino & Brancaccio, 2008). Prospective studies have shown that paricalcitol effectively and safely suppresses PTH in patients with chronic kidney disease (Cheng et al., 2012), dialysis patients and transplant patients (Palya et al., 2009) with minimal effect on serum calcium and phosphorus (Ross et al., 2008; Martin et al., 1998).

*Fibrosis and inflammation*

Several other beneficial effects from VRDAs have been shown in both animal and human studies, as the vitamin D receptor is distributed not only in the bone and parathyroid, but also in other tissues, such as the kidney and the vasculature (Valdivielso et al., 2009; Andress, 2006). Several investigators have indicated that paricalcitol is renoprotective in various experimental models through its anti-inflammatory and antifibrotic actions (Tan et al., 2008; Tan et al., 2009; Mizobuchi et al., 2007; Schwarz et al., 1998). Park et al have recently showed that paricalcitol attenuates cyclosporine-induced kidney injury in rat model. (Park et al., 2010). They suggested that paricalcitol exerted an anti-inflammatory and antifibrotic effect via the inhibition of TGF-β1 signalling and thus attenuated the CsA-induced renal tubular cell apoptosis and fibrosis. They furthermore indicated that paricalcitol might be a possible renoprotective agent in a clinical setting.

In renal transplant recipients these effects may be advantageous, as preserving kidney function and reducing cardiovascular risk, is essential in the long-term care of the renal transplant recipient (Fellstrom et al., 2005b).

*Proteinuria*

Persistent proteinuria after renal transplantation is a strong and independent risk factor for renal graft failure, cardiovascular morbidity and mortality (Fellstrom et al., 2005a; Halimi et al., 2007). Both pre-clinical and clinical trials have shown that treatment with paricalcitol is associated with reduction in proteinuria (Fishbane et al., 2009; de et al., 2010; Freundlich et al., 2008; Agarwal et al., 2005; Alborzi et al., 2008). In the recently published Vital study in patients with type 2 diabetic nephropathy there was a significant reduction of proteinuria with 2 μg paricalcitol, but not with 1 μg daily (de et al., 2010). Whether this effect of paricalcitol also is present in renal transplant recipients is unknown and needs to be investigated.

*Vascular calcification*

It is suggested that coronary artery calcification and the elastic properties of aorta and large arteries are related processes (van Popele et al., 2006). The elastic property of the aorta may be assessed by measuring carotid-femoral pulse wave velocity (PWV), and aortic stiffness has been associated with atherosclerosis of the coronary arteries in older adults (van Popele et al., 2006; Weber et al., 2004). Renal transplant recipients have increased PWV, i.e. aortic stiffness, compared to a control population (Verbeke et al., 2007). We have recently shown that PWV was an independent marker for coronary artery calcification in SLE transplanted patients (Norby et al., 2011). PWV is a strong predictor of all-cause mortality in this population (Mitchell et al., 2010). Paricalcitol may have a superior protect effect on vascular calcification compared to other vitamin D analogs (Cardus et al., 2007; Noonan et al., 2008; Rodriguez et al., 2011).

In summary, paricalcitol may be beneficial for kidney transplant recipients, both in treating persistent hyperparathyroidism and bone disease and cardiovascular effects. However, the most intriguing aspect by paricalcitol treatment is the potential to prevent fibrosis in the transplanted kidney and to reduce proteinuria in this patient population. To date, the effects of paricalcitol in renal transplant recipients are largely unknown, and no randomized controlled data exists in this population.

# OBJECTIVES

Primary objective:

The primary aim of this study is to address the effect of paricalcitol on albumin excretion in renal transplant recipients.

Secondary objectives:

Important secondary endpoints are the effect of paricalcitol on:

- development of graft fibrosis and markers for fibrosis
- glomerular filtration rate
- Bone density
- Pulse wave velocity

# POPULATION

## Inclusion Criteria

In order to participate in this study the patients must meet all of the following inclusion criteria:

1. Patients over the age of 18, who have received a kidney transplant.
2. Patients with a functioning kidney transplant, defined as eGFR>30 ml/min.
3. Patient on triple immunosuppression with CNI inhibitor (cyclosporine or tacrolimus), mycophenolate mofetil or mycophenolate acid, and prednisolone.
4. Serum calcium between 2.0 and 2.6 mmol/l
5. Signed informed consent.

## Exclusion Criteria

In order to participate in the study patients must not meet any of the following exclusion criteria:

1. Patients with tertiary hyperparathyroidism previously operated with parathyroidectomy.
2. Patients treated with vitamin-D analogues after transplantation.
3. Patients who received a deceased donor kidney, from a donor >75 years.
4. Patients with a history of an allergic reaction or significant sensitivity to paricalcitol or drugs similar to study drug.
5. Use of calcimimetics after transplantation and throughout the study period.

## Recruitment and Screening

Patients will be recruited from the nephrology wards participating in the study. Patients who are considered eligible by the Investigator will have the study explained to them, and will receive the written patient information sheet (PIS). After having had the time to review the nature of the study, they will have the opportunity to ask questions to the investigational team. If, after this, the patients agree to participate, they will be asked to sign and date one original of the written informed consent form (ICF). The patients will then receive a copy of the signed and dated patient information/informed consent form. The original signed ICF will be filed in the Investigator Site File (ISF). The PIS will contain site contact information in case of any questions or medical emergency.

Any written information given to potential patients will be submitted to, and approved by, the respective Ethics Committee(s) prior to implementation.

The Investigator will maintain a Patient Screening Log to collect information on all patients who sign an ICF regardless of whether or not they meet the study eligibility criteria following completion of the screening evaluations. After completion of Screening, all patients deemed eligible to take part in this study will be entered onto an Enrolment Log.

## Patient Withdrawal

In accordance with the Declaration of Helsinki, the Investigator must explain to the patient that they have the right to withdraw from the study at any time, and that this will in no way prejudice their future treatment. However, unless safety issues occur, we plan to follow the patients for the entire duration of the study in order to analyse efficacy and safety variables also for those patients withdrawing from the study. The reason for any kind of withdrawal must be recorded on the appropriate CRF.

There will be two main categories for withdrawals from the study:

Complete withdrawal:

Stopping investigational product and also continued efficacy and safety evaluations

Standard reasons for withdrawing from further participation in the study and from the follow-up visits

- Patient’s decision (withdrawal of consent to participate)
- Patient lost to follow-up

Withdrawals from investigational product:

Stopping investigational product, but continuing follow-up visits, including efficacy and safety evaluations

Standard reasons for withdrawing from taking further investigational product, but continuing follow-up visits and safety evaluations may be:

- Unacceptable adverse events
- Patient request
- Investigator's discretion
- Patient lost to follow-up/non-attendance
- Intercurrent illness

However, whenever a patient is withdrawn from a study, or for whatever reason is not coming to any further visits, a final study evaluation must be completed for that patient (visit <>) - stating the reason(s) why the patient was withdrawn from the study. All documentation concerning the patient must be as complete as possible. Every effort will be made in order to follow-up and complete patient evaluation. Patients who are withdrawn for any reason will also be evaluated according to the evaluation flow-chart.

Withdrawals due to non-attendance must be followed up by the Investigator to obtain the reason for non-attendance. Withdrawals due to intercurrent illnesses or adverse events must be fully documented in the case record form, with the addition of supplementary information if available and/or appropriate.

# TREATMENT PROCEDURES

## Overall Study Design

The study is to be performed as a randomized open intervention study. Patient eligibility for the trial will be assessed around 4 weeks after transplantation. If the patient is considered eligible by the Investigator, the patient will attend the Baseline visit (Visit 1, Day 0). At the Baseline visit, the patient will undergo the study-specific evaluations listed in Section 4.3 and will be randomized in a 1:1 ratio to either treatment with paricalcitol or no treatment based on a computer-based allocation sequence.

After the Baseline visit has been completed, the patient will be asked to return to the clinic for the Follow-up visits in the study. The initial Follow-up visits will be the Initial Safety Follow-up visits held every two weeks after the Baseline visit up to Week 6. During these visits to the clinic, blood samples will be taken from the patient for laboratory evaluations and new or changes to pre-existing adverse events will be recorded. After Week 6 up to the final Follow-up visit held at Week 48, the patients will be followed up as per standard clinical practice.

At Week 48 of the study, the patient will return to the clinic for the final Final Follow-up visit (Visit 12, Week 48). This visit will evaluate both efficacy and safety parameters, further listed and described in Section 4.3/Section and Section 4.2, respectively. Every effort will be made in order to follow-up and complete patient evaluation at these Follow-up visits. Patients who are withdrawn for any reason will also be evaluated according to the evaluation flow-chart if possible.

## Assessments and Procedures

### Clinic visits

Visits to the clinic will be performed at Baseline (Visit 2, Day 0), Initial Safety Follow-up (Visit 3a-3c, Week 2/4/6) and at the Final Safety Follow-up (Visit 12, Week 48).

The Screening visit shall be held approximately 4 weeks after the transplantation procedure is completed.

### Informed consent

All subjects participating in the study will be required to be thoroughly informed of the study, given the opportunity to ask study-related questions to the Investigator at the site, and must be given sufficient time to review the patient information sheet. Following this procedure, all subjects willing to participate in the study will be asked to sign and date the informed consent form. The Investigator will also sign and date the form to corroborate that the subject has been thoroughly informed about the study. A photocopy of the signed form will be created by the site who will distribute the copy to the patient. The original informed consent is to be retained by the site.

### Review of inclusion/exclusion criteria

During the Screening Visit Investigator will perform a thorough review of the eligibility criteria for the study. The subject is required to fulfill all of the inclusion criteria and must not fulfill any of the exclusion criteria to be eligible for inclusion in the study. Patients who are considered eligible will also be re-reviewed with regard to eligibility at the Baseline Visit.

### Medical history and concomitant diseases

A review of the subject’s medical history and concomitant diseases will be performed at Screening and at Baseline by the Investigator asking the subject for any information and/or by the Investigator reviewing the patient file if this information is available. A complete review of the subject’s medical history and concomitant diseases will be undertaken by the Investigator to ensure that all inclusion and no exclusion criteria have been met. The Investigator may, with the consent of the subject, contact other physicians who may be or have been responsible for the medical care of the subject in order to establish relevant medical history.

Any medical history considered relevant for the study by the Investigator, including all items listed below, shall be reported. Any conditions which have not presented within the last 5 years shall not be reported. .

All currently active concomitant diseases, whether considered relevant for the study or not by the Investigator, must be reported. The duration of diagnosis of medical history and concomitant diseases should be noted.

In particular, the review should determine whether any of the following conditions are currently present or have been in the past:

Hypercalcemia and/or treated by Mimpara

### Concomitant medication

See Section 5.5, below.

### Physical examination

The physical evaluation will include a review of organ systems by the Investigator as per standard clinical practice.

### Vital signs

The following vital sign evaluations will be performed at various visits during the study and recorded:

- Blood pressure: Will be performed as seated (after at least 5 minutes of rest) systolic and diastolic blood pressure (in mmHg).

All new findings or changes to previous findings considered clinically significant are to be recorded in the medical journal

### Randomisation

After review and confirmation that all study eligibility criteria have been met, the Investigator will assign a randomisation number to the subject on the Baseline Visit.

Additional information on the randomization procedure is available in Section 5.4.

### Study drug administration.

Patients allocated to Paricalcitol will get a prescription of orally 2 ug once daily for the entire duration of the study, unless reduction to 1 ug once a day due to hypercalcamia.

Compliance will be monitored with counting of capsules and collection of blister packs.

### Efficacy evaluation methods

#### Proteinuria

Measured as albumin/creatinine ratio at baseline and after 12 months.

**Note:** Proteinuria (albumin/creatinine ratio) will be measured automatically for the Baseline (Visit 1, Day 0) and Final Safety Follow-up (Visit 4, Week 48).

#### Kidney biopsy

A kidney biopsy will be performed at the Baseline visit (and at the Final Safety Follow-up in order to evaluate the degree of fibrosis, according to IF/TA (interstitial fibrosis/tubular atrophy). Kidney biopsy will be performed by the department of radiology according standard technique and will be evaluated according to degree of IF/TA. One section of the biopsy will be utilized to perform assessment of markers of fibrosis.

**Protocol biopsies are routinely performed on all transplanted patients at week 6 and month 12.**

Two renal biopsies are mandatory in the study protocol. The first biopsy should be taken at the time of inclusion, the second at 12 months after transplantation, respectively. There will not be required to obtain extra biopsy due to participation in the trial.

Biopsies will be taken according to our center’s common routine.

The Banff standard regarding the presence of sufficient number of glomeruli and arteries will be used

The laboratory will stain their own according to local practice. In addition the slides will be stained for C4d. The routine biopsy for C4d will be utilized for expression of fibrosis markers.

#### Expression of Fibrosis markers in graft biopsies

Graft fibrosis will be assessed by Banff criteria and by morphological techniques.

RNA expression of following fibrosis markers

Transforming growth factor beta (TGF-beta)

Osteopontin

Thrombospondins

Tenascins

Osteonectin/Sparcs

Periostin

#### Clinical laboratory tests - blood

At all visits to the clinic routine laboratory samples are to be taken for the assessment of haematological and clinical chemistry variables.

The following variables will be measured:

Haematology:

[WBC](http://www.labtestsonline.org/understanding/analytes/glucose/glance.html) (white blood cells), [HgB](http://www.labtestsonline.org/understanding/analytes/glucose/glance.html) (haemoglobin), and platelets.

Clinical chemistry:

[Calcium](http://www.labtestsonline.org/understanding/analytes/calcium/glance.html), sodium, potassium, creatinine, AST (aspartate aminotransferase), ALT (alanine aminotransferase), BASP, LD, bilirubin, albumin, blood urea nitrogen (BUN), parathyroid hormone (PTH) and C-reactive protein (CRP).

#### Clinical laboratory tests - urine

Proteinuria will be evaluated with a spot urine for albumin/creatinine. Spot urine will be measured according to standard methods. Urine samples for evaluation of proteinuria will be taken at Baseline and at the Final Safety Follow-up visit.

#### Markers of Bone Turnover and Inflammation status

- PTH-3 generations assay
- 25-vit D
- PINP (N-terminal propeptide of human procollagen type I)
- Osteocalcin
- NTX (cross-linked N-telopeptides of bone type I collagen)
- Fasting blood glucose
- Insulin
- Leptin
- Adiponectin Fetuin A
- BAP (bone specific alkaline phosphatase)
- FGF23
- Calcitonin
- Osteoprotegerin
- Selected biomarkers reflecting inflammatory pathways (to be determined later)
- Body composition (by BMD).

#### Markers of Crystallization inhibitors

Fetuin-A, MGP and FGF-23.

#### Pulse Wave Velocity

1. Arterial stiffness will be assessed by means of PWV measurements using “SphygmoCor™” (Atcor Medical, Sidney, Australia), a non-invasive technique with direct-contact pulse sensors (Covic et al., 2005). Pulse waveforms will be recorded simultaneously, obtained transcutaneously over the common carotid artery and femoral artery in the groin. The PWV will be calculated as the distance between recording sites measured over the surface of the body (L), divided by the time interval (t) between the feet of the flow waves (PWV = L/t). The value is averaged over 10 cardiac cycles (Asmar et al., 1995).

#### Bone Mineral Density

Standard clinical measurements.

#### Glomerular filtration rate estimated by Modification of Diet of Renal Disease (MDDR) formula.

Kidney function is to be evaluated with estimated GFR (eGFR) at the Baseline visit (Visit 2, Day 0) and at the Final Safety Follow-up visit (Visit 4, Week 48).

### Safety evaluation methods

During the course of the trial the investigator will report adverse events. Adverse events including intensity, duration and relation to treatment will be recorded in the Case Report Form (CRF). Adverse events and serious adverse events will be collected from the date of informed consent until 7 days after the last dose of the investigational product. All adverse events will be followed up until resolved or as clinically required. For details about adverse event reporting, please see Section 6.3, below.

Safety data will be monitored throughout the study and regularly reviewed by an independent Data Monitoring Committee (DMC).

## Scheduling of Procedures

### Screening

- Informed consent
- Inclusion/exclusion criteria review
- Medical history and concomitant diseases
- Concomitant medication
- Physical examination
- Vital signs

### Visit 1, Baseline (Day 0) (week 6 after transplantation)

- Review of eligibility criteria
- Review of medical history and concomitant diseases
- Concomitant medication
- Physical examination
- Vital signs
- Randomisation
- Study drug administration
- Estimated GFR
- Kidney biopsy
- Clinical laboratory tests
- Urine samples
- Markers of bone turnover
- Endothelial- and inflammatory markers
- Echocardiography
- Pulse wave velocity
- Bone mineral density
- Adverse events

### Visits 3a-3c, Initial Safety Follow-up (Week 2, 4 and 6)

- Concomitant medication
- Clinical laboratory tests
- Adverse events

### Visit 10, Final Safety Follow-up (Week 48)

- Concomitant medication
- Physical examination
- Vital signs
- Clinical laboratory tests
- Urine samples
- Markers of bone turnover
- Endothelial- and inflammatory markers
- CD19
- Immunoglobulins
- DSA
- Estimated GFR
- Kidney biopsy
- Adverse events

# STUDY MEDICATION

## Study Medication and Comparator

Patients are randomized into two arms. Paricalcitol will be prescribed for the active arm.

## Dosage and Administration

### *Paricalcitol Titration*

The dose for paricalcitol will be 2 μg/day. The data monitoring committee will monitor levels of Ca, P and PTH. The dose of paricalcitol should be reduced with 50% if s-calcium exceeds 2.75 (11 mg/dl) at 2 consecutive measurements.

## Duration of Treatment

Patient will be receiving treatment for 48 months after randomization.

## Methods for Assigning Patients to Treatment

All patients signing the ICF and who enter the formal screening processwill be assigned a unique screening number.

Following successful completion of the screening/baseline evaluations and confirmation that the patient is eligible for participation, the patient will be randomised to treatment or not. This will be performed by the Investigator assigning a randomization number to the patient

## Concomitant Medication

Any medication (prescription as well as over the counter (OTC) drugs) or therapeutic intervention deemed necessary for the patient, and which, in the opinion of the Investigator, do not interfere with the safety and efficacy evaluations, may be continued unless they are included in the list of *‘medications and therapeutic regimens excluded from the study’* outlined below.

Any new medications or changes to the dose or regimen of pre-existing medications will be updated on a routine basis during the study.

Investigational new drugs (i.e. drugs that are not marketed in the local market) should not be co-administered with the study medications during the entire period of the study.

## Study Medication Accountability

Compliance will be monitored with counting of capsules and collection of blister packs.

# RESPONSE VARIABLES AND ENDPOINTS

## Assessment of Efficacy

### Primary Efficacy end-point

The primary end-point is reduction of albumin excretion

### Secondary Efficacy Variables

Secondary efficacy variables will include:

- Proteinuria, measured with albumin/creatinine ratio.
- Fibrosis, evaluated with Interstitial fibrosis/Tubular Atrophy (IF/TA), and expression of fibrosis markers in a kidney biopsy after 12 months
- Reduction in PTH
- Reduction in pulse wave velocity
- Increase in bone mineral density
- Renal function measured assessed by estimated GFR
- Change in circulating inflammatory biomarkers
- Change in biomarkers of bone turnover.

## Assessment of Safety

### Study-specific Safety Variables

If an unexpected, clinically significant change from baseline occurs in a laboratory value or other measurement, the Investigator should repeat the test if appropriate, to verify the result. The Investigator must comment on all clinically significant changes in laboratory values. All clinically significant adverse events must be followed-up and this follow-up documented until the events return to normal or become stabilised.

## Adverse Events and Serious Adverse Events

### Definitions of Adverse Events

#### Seriousness

Adverse Events (AE):

Any undesirable experience occurring to a patient during a clinical study, whether or not considered related to the investigational study medication(s).

All Adverse Events must be recorded in the case report form, defining relationship to study medication and severity. Adverse Events should also be recorded by the Investigator in the patient file/notes.

Serious Adverse Events (SAE):

A serious adverse event (experience) or reaction is any untoward medical occurrence that at any dose results in death, is life-threatening, requires in-patient hospitalisation or prolongation of existing hospitalisation, results in persistent or significant disability/incapacity, or is a congenital anomaly/birth defect, is a medically important event or reaction.

**Note:** The term "life-threatening" in the definition of "serious" refers to an event in which the patient was at risk of death at the time of the event; it does not refer to an event which hypothetically might have caused death if it were more severe.

Medical and scientific judgement should be exercised in deciding whether expedited reporting is appropriate in other situations, such as important medical events that may not be immediately life-threatening or result in death or hospitalisation but may jeopardise the patient or may require intervention to prevent one of the other outcomes listed in the definition above. These should also usually be considered serious.

Unexpected Adverse Event:

An experience not previously reported in the Summary of Products Characteristics (SPC).

#### Severity

Mild:

The adverse event is transient and easily tolerated.

Moderate:

The adverse event causes the patient discomfort and interrupts the patient's usual activities.

Severe:

The adverse event causes considerable interference with the patient's usual activities, and may be incapacitating or life-threatening.

#### Relationship to study medication

Unrelated:

This category is applicable to those adverse events which, after careful medical consideration at the time of evaluation, are judged to be clearly and incontrovertibly due to extraneous causes (disease, environment, etc.) and do not meet the criteria for study medication relationship listed under remote, possible or probable.

Unlikely:

In general, this category is applicable to those adverse events that, after careful medical consideration at the time they are evaluated, are judged to be remotely related to the test study medication. An adverse event may be considered remote if, or when, for example:

1. It does not follow a reasonable temporal sequence from administration
2. It could readily have been produced by the patient's clinical state, environmental or toxic factors, or other modes of therapy administered to the patient
3. It does not follow a known response pattern to the suspected study medication
4. It does not reappear or worsen when the study medication is re-administered

Possible: *(must have first two criteria)*

This category is applicable to those adverse events which, after careful medical consideration at the time they are evaluated, the connection with the test study medication administration appears unlikely but cannot be ruled out with certainty. An adverse event may be considered possible if, or when:

1. It follows a reasonable temporal sequence from administration
2. It could readily have been produced by the patient's clinical state, environmental or toxic factors, or other modes of therapy administered to the patient
3. It follows a known response pattern to the suspected study medication
4. It does not reappear or worsen when the study medication is re-administered

Probable: *(must have first three criteria)*

This category is applicable to those adverse events, which, after careful medical consideration at the time they are evaluated, the connection with the test study medication administration appears to, with a high degree of certainty, be related to the test study medication. An adverse event may be considered probable if:

1. It follows a reasonable temporal sequence from administration
2. It could not be reasonably explained by the known characteristics of the patient's clinical state, environmental or toxic factors, or other modes of therapy administered to the patient
3. It disappears or decreases on cessation or reduction in dose. There are important exceptions when an adverse event does not disappear upon discontinuation of the study medication, yet study medication-relatedness exists; e.g. bone marrow depression, fixed study medication eruptions, and tardive dyskinesias
4. It follows a known response pattern to the suspected study medication
5. It reappears upon rechallenge

Not assessable:

When it is not possible to assign the event to any of the criteria above categories.

### Reporting of adverse events

All Adverse Events must be recorded in the case report form, defining relationship to study medication and severity.

**As soon as the Investigator is aware of a potential Serious Adverse Event (SAE), he/she should contact by phone, fax or e-mail, and in any case no later than 24 hours after the knowledge of such a case**.

At the time of the call, the Investigator must provide as a minimum requirement, the patient number, birth date, nature of the SAE, and a preliminary assessment of causality. The Investigator should follow-up the initial notification of the potential SAE by faxing a copy of the SAE reporting form to **Renal Section, Department of Transplant Medicine**

Follow-up information on an existing SAE that is fatal or life-threatening should be reported by the Investigator **Renal Section, Department of Transplant Medicine**  within 5 days after the initial report. Where appropriate, hospitalisation or autopsy reports should be made available. All Serious Adverse Events will be followed up until resolution (i.e., asymptomatic, stabilisation or death).

## Serious Adverse Reactions and Unexpected Adverse Reactions

### Definitions

Adverse Reaction:

All noxious and unintended responses to a medicinal product related to any dose should be considered adverse drug reactions.

The phrase "responses to a medicinal product" means that a causal relationship between a medicinal product and an adverse event is at least a reasonable possibility, i.e., the relationship cannot be ruled out.

For marketed medicinal products, an adverse reaction is a response to a drug which is noxious and unintended and which occurs at doses normally used in man for prophylaxis, diagnosis, or therapy of disease or for modification of physiological function.

Unexpected Adverse Reaction:

An adverse reaction, the nature or severity of which is not consistent with the applicable product information (e.g. product information sheet such as the Summary of Products Characteristics (SPC)).

Suspected Unexpected Serious Adverse Reaction (SUSAR):

Any serious adverse reaction that might be related to the study medication and are unexpected according to the definition above.

### Reporting of suspected unexpected serious adverse reactions by CRO

Suspected unexpected serious adverse reactions (SUSARs) will be reported **Renal Section, Department of Transplant Medicine** according to appropriate Competent Authority and Ethics Committee requirements. CRO will report SUSARs to Investigators <preferably twice yearly, should be adapted to the type of trial according to ICH Good Clinical Practice and to local regulations. SUSAR reporting to the Competent Authorities and Ethics Committees will be performed according to local regulations. The Competent Authorities will be notified of all SUSARs through the Eudravigilance database.

Fatal and life-threatening SUSARs should be reported by **Renal Section, Department of Transplant Medicine** as soon as possible to the Competent Authorities and Ethics Committees according to local regulations, and in any case no later than seven calendar days, after knowledge by CRO of such a case. Relevant follow-up information on the case will be subsequently communicated within an additional eight days. All other SUSARs shall be reported to the Competent Authorities concerned and to the Ethics Committee concerned according to local regulations as soon as possible but within a maximum of fifteen days of first knowledge by **Renal Section, Department of Transplant Medicine.**

# STATISTICAL METHODOLOGY AND DATA MANAGEMENT

## Study Design

This clinical trial employs a Randomized, open control design. Randomisation is used to minimise assignment bias and to increase the likelihood that known and unknown patient attributes. Estimation of Sample Size:

Assuming a type 1 error of 5% and at type II error of 20 %, with a clinically relevant difference in 5.2 mg/mmol from a baseline value of 15.0 + 10 mg/mmol the estimated number of patients in each arm should be 30, assuming a correlation between start and end value of 0.5. In case of early drop-outs additional patients can be included to ensure that a total number of 70 patients will be randomized.

## Randomisation

The randomisation scheme (in a 1.1 ratio) will be generated by CRO using a computerised procedure. A complete randomisation list containing details of all patient numbers and study group will be stored as essential documentation in the Trial Master File and at the study site (open study).

## Statistical Analysis Plan

The main hypothesis:

In renal transplant recipients’ treatment with paricalcitol is superior to placebo, and results in reduced albuminuria at 12 months treatment.

## Study Populations

Intention-to-treat population:

The ITT population will consist of all randomized patients who received at least one dose of active medication or placebo therapy after Visit 1 and have at least one post-baseline assessment of the primary efficacy variable (proteinuria). Randomized patients without data on the primary outcome variable will be excluded from this population.

The ITT population will be analyzed following the intent to treat principle.

Per protocol population:

The Per-protocol Population will consist of all ITT patients who did not show major deviations from the protocol procedures that may have an impact on the study outcome and who have completed the treatment phase at 12 months according to protocol. Criteria that are assumed to have such an impact will be defined before analysis during the Blind Review Meeting.

Safety population:

The Safety Population (SAF population) will consist of all patients in whom transplantation was performed and who were randomized and treated with at least one dose of randomized treatment

## Data Collection / Case Report Forms

Data will be entered and collected from journal and lab forms. All data must be stored in an unidentifable form treated with strict confidentiality in accordance with applicable data-protection regulations.

Case report forms (CRFs) will be supplied for recording data from each patient. It is the responsibility of the Investigator to ensure that these case report forms are properly completed. The Investigator will sign the designated signature pages to confirm that the case report form is accurate and complete.

To ensure legibility the CRFs should be completed in block capitals with a black or blue ballpoint pen (not pencil, felt-tip or fountain pen).

Any corrections to the CRFs must be carried out by the Investigator or his designate. A single stroke must be drawn through the original entry. The correction has to be dated and initialled. Incorrect entries must not be covered with correcting fluid, or obliterated, or made illegible in any way.

Even if there are no changes from a previous examination, in the interests of completeness of data acquisition the questions, which are repeated in each section of the CRFs should be answered in full. A reasonable explanation must be given by the Investigator for all missing data.

The CRFs will be completed and - after being signed by the Investigator - will be collected by a monitor from **Renal Section, Department of Transplant Medicine**.

## Data Management

Data will be transmitted electronically to a research data base at OUS.

# REGULATORY AND ADMINISTRATIVE PROCEDURES

## Institutional Review

The study must be approved by applicable ethics committees, and will be conducted in accordance with the Seoul, 2008 amendment to the Declaration of Helsinki 1964.

The Protocol and the Patient Information Sheet / Informed Consent Form will be approved by the relevant Competent Authorities and Ethics Committees, and possibly other public bodies according to local requirements before commencement. If a protocol amendment is necessary, this will be prepared with the agreement of the Principal Investigator, and signed by the relevant parties. If the amendment is considered to be substantial, it will be submitted to the Competent Authorities and Ethics Committees, and possibly other public bodies according to local requirements for review and approval. The protocol amendment will not be implemented before the required approvals are obtained. Minor amendments which do not affect the safety or physical or mental integrity of the clinical trial participants or the scientific value of the trial (i.e. non-substantial amendments) will not be submitted to Competent Authorities or Ethics Committees.

SUSAR reports and Periodic Safety Reports will be sent to Competent Authorities and Ethics Committees according to local regulations. Registration of data will be carried out according to national data laws.

## Patient Information / Informed Consent

The Investigator is responsible for giving the study patient full and adequate verbal and written information about the nature, purpose, possible risk and benefit of the study. Study patients must also be notified that they are free to withdraw from the study at any time. The patients should have reasonable time to read and understand the information before signing. If a signed consent is impossible to achieve, an oral consent may be sufficient if witnessed by an independent person. The reason for not obtaining a signed consent must be explained by the Investigator. The Investigator is responsible for obtaining signed or oral informed consent from all patients before including the patient in any study related procedures.

A signed copy of the patient information and of the Informed Consent Form in local language, will be given to the patients.

## Patient Confidentiality

The Investigator must ensure that patient’s confidentiality will be maintained

The Investigator should keep a separate log of patient codes and names

The Investigator is required to record primary efficacy and safety data, concomitant medication and patient progress in the patient’s file/notes/medical record.

## Patient Treatment Plan

After the individual completion of the study, the patient will return to the standard treatment received prior to study participation

## GCP

The study will be managed and conducted according to the latest International Conference on Harmonisation (ICH) guidelines for Good Clinical Practice (GCP).

## Essential Documents

The ICH guideline for GCP lists a number of essential GCP documents required prior to, during and after the conduct of the study. It is the responsibility of the monitor to ensure that the Investigator is always provided with a copy of such documents prepared by the study management, and it is likewise the responsibility of the Investigator to provide the monitor with essential documents prepared by the Investigator or the local Ethics Committee. A complete list of essential GCP documents can be found in the Investigator Site File.

## Record Retention

All medical records upon which the clinical data are based (source data) must be kept for at least 5 years or according to local legislation whichever is the longest after completion of the study. Image carriers or other data carriers may be used for this purpose. The documentation should be easily retrievable and readable during the entire archiving duration.

## Quality Assurance

During or after the study is completed, regulatory authorities may wish to carry out an audit or an inspection.

## Insurance and Liability

Liability for study medication-induced injury will be according to local requirements and in accordance to national regulation.

## End of Trial

The end of the trial is defined as the last visit of the last patient included in the trial. The Competent Authorities and the Ethics Committees, as applicable, will be notified about the end of the trial.

## Study Report

A clinical study report (CSR) will be prepared covering clinical and statistical aspects and summarising all findings of the clinical study. The content has to be treated as strictly confidential. The study report will be sent to the Investigators, the Competent Authorities and Ethics Committees according to local requirements.

## Publication and Data Rights

The study will be registered in www.clinicaltrials.gov.

It is envisaged that the findings of the study will, in due course and by mutual agreement, be published in a scientific journal and/or presented at a scientific meeting. The authorship of this publication will remain as specified below, including at least the principal Investigator and a representative for CRO; all participating Investigators will be mentioned and listed as members of the study group.

The published international guidelines for authorship (International Committee of Medical Journal Editors, 1997) will be adhered to; i.e. ‘All persons designed as authors should qualify for authorship. Each author should have participated sufficiently in the work to take public responsibility for the content.’

Authorship credit will therefore be based only on substantial contributions to 1) conception and design, or analysis and interpretation of data; and to 2) drafting the article or revising it critically for important intellectual content; and on 3) final approval of the version to be published. Conditions 1), 2) and 3) must all be met. Participation solely in acquisition of funding or the collation of data does not justify authorship. General supervision of the research group is not sufficient for authorship. It is intended that information on what each author has contributed will be published.

It is emphasised however, that only those who entirely meet the above mentioned criteria will be listed as authors.

# REFERENCES

Reference List

Agarwal, R., Acharya, M., Tian, J., Hippensteel, R. L., Melnick, J. Z., Qiu, P. et al. (2005). Antiproteinuric effect of oral paricalcitol in chronic kidney disease. *Kidney International, 68,* 2823-2828.

Alborzi, P., Patel, N. A., Peterson, C., Bills, J. E., Bekele, D. M., Bunaye, Z. et al. (2008). Paricalcitol reduces albuminuria and inflammation in chronic kidney disease: a randomized double-blind pilot trial. *Hypertension, 52,* 249-255.

Andress, D. L. (2006). Vitamin D in chronic kidney disease: a systemic role for selective vitamin D receptor activation. *Kidney International, 69,* 33-43.

Asberg, A., Midtvedt, K., Line, P. D., Narverud, J., Holdaas, H., Jenssen, T. et al. (2006). Calcineurin inhibitor avoidance with daclizumab, mycophenolate mofetil, and prednisolone in DR-matched de novo kidney transplant recipients. *Transplantation, 82,* 62-68.

Asmar, R., Benetos, A., Topouchian, J., Laurent, P., Pannier, B., Brisac, A. M. et al. (1995). Assessment of arterial distensibility by automatic pulse wave velocity measurement. Validation and clinical application studies. *Hypertension, 26,* 485-490.

Cardus, A., Panizo, S., Parisi, E., Fernandez, E., & Valdivielso, J. M. (2007). Differential effects of vitamin D analogs on vascular calcification. *Journal of Bone and Mineral Research, 22,* 860-866.

Chapman, J. R., O'Connell, P. J., & Nankivell, B. J. (2005). Chronic renal allograft dysfunction. *Journal of the American Society of Nephrology, 16,* 3015-3026.

Cheng, J., Zhang, W., Zhang, X., LI, X., Chen, J. (2012). Efficacy and safety of paricalcitol therapy for chronic kidney disease: A meta-analysis *Clinical Journal of the American Society of Nephrology, epub, ahead of print.*

Covic, A., Gusbeth-Tatomir, P., & Goldsmith, D. J. (2005). Arterial stiffness in renal patients: an update. *American Journal of Kidney Diseases, 45,* 965-977.

Cozzolino, M. & Brancaccio, D. (2008). Emerging role for the vitamin D receptor activator (VDRA), paricalcitol, in the treatment of secondary hyperparathyroidism. *Expert.Opin.Pharmacother., 9,* 947-954.

de, Z. D., Agarwal, R., Amdahl, M., Audhya, P., Coyne, D., Garimella, T. et al. (2010). Selective vitamin D receptor activation with paricalcitol for reduction of albuminuria in patients with type 2 diabetes (VITAL study): a randomised controlled trial. *Lancet, 376,* 1543-1551.

Fellstrom, B., Holdaas, H., Jardine, A. G., Nyberg, G., Gronhagen-Riska, C., Madsen, S. et al. (2005a). Risk factors for reaching renal endpoints in the assessment of Lescol in renal transplantation (ALERT) trial. *Transplantation, 79,* 205-212.

Fellstrom, B., Jardine, A. G., Soveri, I., Cole, E., Gronhagen-Riska, C., Neumayer, H. H. et al. (2005b). Renal dysfunction as a risk factor for mortality and cardiovascular disease in renal transplantation: experience from the Assessment of Lescol in Renal Transplantation trial. *Transplantation, 79,* 1160-1163.

Fishbane, S., Chittineni, H., Packman, M., Dutka, P., Ali, N., & Durie, N. (2009). Oral paricalcitol in the treatment of patients with CKD and proteinuria: a randomized trial. *American Journal of Kidney Diseases, 54,* 647-652.

Freundlich, M., Quiroz, Y., Zhang, Z., Zhang, Y., Bravo, Y., Weisinger, J. R. et al. (2008). Suppression of renin-angiotensin gene expression in the kidney by paricalcitol. *Kidney International, 74,* 1394-1402.

Halimi, J. M., Buchler, M., Al-Najjar, A., Laouad, I., Chatelet, V., Marliere, J. F. et al. (2007). Urinary albumin excretion and the risk of graft loss and death in proteinuric and non-proteinuric renal transplant recipients. *Am.J.Transplant., 7,* 618-625.

Hazzan, M., Hertig, A., Buob, D., Copin, M. C., Noel, C., Rondeau, E. et al. (2011). Epithelial-to-Mesenchymal Transition Predicts Cyclosporine Nephrotoxicity in Renal Transplant Recipients. *Journal of the American Society of Nephrology, 22,* 1375-1381.

Johnson, D. W., Saunders, H. J., Johnson, F. J., Huq, S. O., Field, M. J., & Pollock, C. A. (1999). Cyclosporin exerts a direct fibrogenic effect on human tubulointerstitial cells: roles of insulin-like growth factor I, transforming growth factor beta1, and platelet-derived growth factor. *Journal of Pharmacology and Experimental Therapeutics, 289,* 535-542.

Martin, K. J., Gonzalez, E. A., Gellens, M., Hamm, L. L., Abboud, H., & Lindberg, J. (1998). 19-Nor-1-alpha-25-dihydroxyvitamin D2 (Paricalcitol) safely and effectively reduces the levels of intact parathyroid hormone in patients on hemodialysis. *Journal of the American Society of Nephrology, 9,* 1427-1432.

Mitchell, A., Opazo, S. A., Kos, M., Witzke, O., Kribben, A., & Nurnberger, J. (2010). Pulse wave velocity predicts mortality in renal transplant patients. *European Journal of Medical Research, 15,* 452-455.

Mizobuchi, M., Morrissey, J., Finch, J. L., Martin, D. R., Liapis, H., Akizawa, T. et al. (2007). Combination therapy with an angiotensin-converting enzyme inhibitor and a vitamin D analog suppresses the progression of renal insufficiency in uremic rats. *Journal of the American Society of Nephrology, 18,* 1796-1806.

Myers, B. D., Ross, J., Newton, L., Luetscher, J., & Perlroth, M. (1984). Cyclosporine-associated chronic nephropathy. *New England Journal of Medicine, 311,* 699-705.

Naesens, M., Kuypers, D. R., & Sarwal, M. (2009). Calcineurin inhibitor nephrotoxicity. *Clin.J.Am.Soc.Nephrol., 4,* 481-508.

Nankivell, B. J., Borrows, R. J., Fung, C. L., O'Connell, P. J., Allen, R. D., & Chapman, J. R. (2003). The natural history of chronic allograft nephropathy. *New England Journal of Medicine, 349,* 2326-2333.

Noonan, W., Koch, K., Nakane, M., Ma, J., Dixon, D., Bolin, A. et al. (2008). Differential effects of vitamin D receptor activators on aortic calcification and pulse wave velocity in uraemic rats. *Nephrology, Dialysis, Transplantation, 23,* 3824-3830.

Norby, G. E., Gunther, A., Mjoen, G., Andersen, R., Dolgos, S., Hartmann, A. et al. (2011). Prevalence and risk factors for coronary artery calcification following kidney transplantation for systemic lupus erythematosus. *Rheumatology.(Oxford)*.

Paya, A., Noor, F., Vania, S., Kumar, M., Rangana, K., Ahmed, Z. (2009). Vitamin D analogues reduce proteinuria in kidney transplant (KTx) patients. *Abstract, SpringClinical Meeting of the national kidney foundation; March 25-29, 2009, Nasville TN, USA* .

Park, J. W., Bae, E. H., Kim, I. J., Ma, S. K., Choi, C., Lee, J. et al. (2010). Paricalcitol attenuates cyclosporine-induced kidney injury in rats. *Kidney International, 77,* 1076-1085.

Rodriguez, M., Martinez-Moreno, J. M., Rodriguez-Ortiz, M. E., Munoz-Castaneda, J. R., & Almaden, Y. (2011). Vitamin d and vascular calcification in chronic kidney disease. *Kidney Blood Press Res., 34,* 261-268.

Ross, E. A., Tian, J., Abboud, H., Hippensteel, R., Melnick, J. Z., Pradhan, R. S. et al. (2008). Oral paricalcitol for the treatment of secondary hyperparathyroidism in patients on hemodialysis or peritoneal dialysis. *American Journal of Nephrology, 28,* 97-106.

Schwarz, U., Amann, K., Orth, S. R., Simonaviciene, A., Wessels, S., & Ritz, E. (1998). Effect of 1,25 (OH)2 vitamin D3 on glomerulosclerosis in subtotally nephrectomized rats. *Kidney International, 53,* 1696-1705.

Tan, X., He, W., & Liu, Y. (2009). Combination therapy with paricalcitol and trandolapril reduces renal fibrosis in obstructive nephropathy. *Kidney International, 76,* 1248-1257.

Tan, X., Wen, X., & Liu, Y. (2008). Paricalcitol inhibits renal inflammation by promoting vitamin D receptor-mediated sequestration of NF-kappaB signaling. *Journal of the American Society of Nephrology, 19,* 1741-1752.

Valdivielso, J. M., Cannata-Andia, J., Coll, B., & Fernandez, E. (2009). A new role for vitamin D receptor activation in chronic kidney disease. *Am.J.Physiol Renal Physiol, 297,* F1502-F1509.

van Popele, N. M., Mattace-Raso, F. U., Vliegenthart, R., Grobbee, D. E., Asmar, R., van der Kuip, D. A. et al. (2006). Aortic stiffness is associated with atherosclerosis of the coronary arteries in older adults: the Rotterdam Study. *Journal of Hypertension, 24,* 2371-2376.

Verbeke, F., Van, B. W., Peeters, P., Van Bortel, L. M., & Vanholder, R. C. (2007). Arterial stiffness and wave reflections in renal transplant recipients. *Nephrology, Dialysis, Transplantation, 22,* 3021-3027.

Vieira, J. M., Jr., Noronha, I. L., Malheiros, D. M., & Burdmann, E. A. (1999). Cyclosporine-induced interstitial fibrosis and arteriolar TGF-beta expression with preserved renal blood flow. *Transplantation, 68,* 1746-1753.

Weber, T., Auer, J., O'Rourke, M. F., Kvas, E., Lassnig, E., Berent, R. et al. (2004). Arterial stiffness, wave reflections, and the risk of coronary artery disease. *Circulation, 109,* 184-189.

**10. Study flow chart**

| **Period** | **Baseline** |  |  |  |  |  |  |  |  |  |
| --- | --- | --- | --- | --- | --- | --- | --- | --- | --- | --- |
| Study Month | 0 |  |  |  | 1 | 4 | 5 | 7 | 9 | 12 |
| Study Week | 0 | 1 | 2 | 3 | 4 |  |  |  |  |  |
| Study Day |  |  |  |  |  |  |  |  |  |  |
| Visit Number | 1^A^ | 2 | 3 | 4 | 5 | 6 | 7 | 8 | 9 | 10 |
|  |  |  |  |  |  |  |  |  |  |  |
| **Procedures:** |  |  |  |  |  |  |  |  |  |  |
| Informed Consent | X |  |  |  |  |  |  |  |  |  |
| Selection criteria | X |  |  |  |  |  |  |  |  |  |
| Demographics | X |  |  |  |  |  |  |  |  |  |
| Medical History^E^ and Concomitant Diseases^B^ | X |  |  |  |  |  |  |  |  |  |
| Physical Examination | X |  |  |  |  |  |  |  |  | X |
| Vital Signs | X | X |  |  |  |  |  |  |  | X |
| Randomisation | X |  |  |  |  |  |  |  |  |  |
| Study Drug Dispensation^C^ | X |  |  |  |  |  |  |  |  | X |
| Study Drug Accountability |  |  | X | X | X | X | X | X | X | X |
| Study Drug Return | X |  |  |  |  |  |  |  |  | X |
| Drug concentrations^F^ | X |  | x | x | x | x | x | x | x | x |
| Albumin/creatinine ratio | X |  |  |  |  |  |  |  |  | X |
| Kidney Biopsy^D^ | X |  |  |  |  |  |  |  |  | X |
| Clinical Laboratory Tests – blood^I^ | X | X | X | X | X | X | X | X | X | X |
| Study-specific biomarkers^J^ | X |  |  |  |  |  |  |  |  | X |
| Clinical Laboratory Tests - urine | X | X | X | X | X | X | X | X | X | X |
| Echocardiography | X |  |  |  |  |  |  |  |  | X |
| Pulse Wave Velocity | X |  |  |  |  |  |  |  |  | X |
| Bone Mineral Density | X |  |  |  |  |  |  |  |  | X |
| eGFR | X |  | X | X | X | X |  | X | X | X |
| Adverse Events^B^ |  |  | X | X | X | X | X | X | X | X |
| Post-transplantation complications^H^ | X |  | X | X | X X | X | X | X | X | X |
| Concomitant Medication^G^ | X | X | X | X | X | X | X | X | X | X |

^A^Note: Visit should be held approximately 4 weeks after transplantation

^B^Note: All events occurring prior to study drug administration shall be registered

as Medical History and all event subsequent to study mediation administration shall be classified as adverse events.

^C^Note: The patient will be given study medication for the entire treatment period.

^D^Note: TGF-beta levels will be analysed from the kidney biopsy samples

^E^Note: Including transplantation details

^F^Note: Cyclosporine C0/C2 levels

^G^Note: Specific recording of immunosuppressive medication

^H^Note: Rejection episodes, malignancy, infections.

^I^Note: For fertile women: pregnancy test (β-HCG) at baseline

^J^Note: Bone biomarkers, crystallisation markers

11. Modification in renal disease formula

Calculated value of GFR in ml/min - MDRD formula

For men: GFR = 170 x (serum creatinine ^-0,999^) x (age^-0,176^) x (urea nitrogen ^-0,17^) x
 (albumin^0,318^)

For women: GFR = 170 x (serum creatinine ^-0,999^) x (age^-0,176^) x
 (urea nitrogen ^-0,17^) x (albumin^0,318^) x 0.762

with urea nitrogen = urea / 2.144.

The calculated GFR will be done automatically by entering the different variables into the eCRF.
