# Supplementary figures and images for: Exploring the potential effect of paricalcitol on markers of inflammation in de novo renal transplant recipients
Source: PLoS One. 2020 Dec 16;15(12):e0243759. doi: 10.1371/journal.pone.0243759 (PMC7743930; doi:10.1371/journal.pone.0243759)

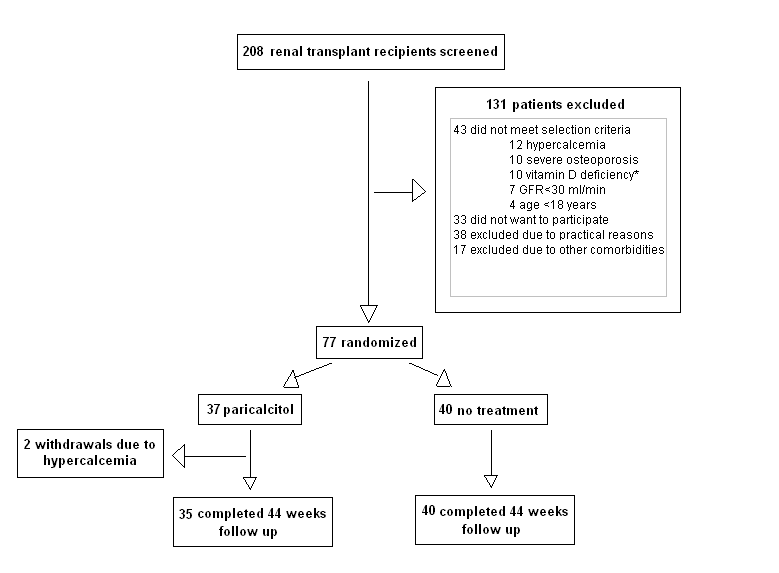

Supplement: S3 File — (BMP) [file pone.0243759.s005.bmp]
