# Supplementary material for: Exploring the potential effect of paricalcitol on markers of inflammation in de novo renal transplant recipients
Source: PLoS One. 2020 Dec 16;15(12):e0243759. doi: 10.1371/journal.pone.0243759 (PMC7743930; doi:10.1371/journal.pone.0243759)
Supplement: Attachment — Submitted filename: PONE-D-20-18424_reviewer.pdf [file pone.0243759.s006.pdf]

PLOS ONE

Exploring the potential effect of paricalcitol on markers of inflammation in de novo renal transplant recipients

--Manuscript Draft--

**Manuscript Number:**	PONE-D-20-18424
**Article Type:**	Research Article
**Full Title:**	Exploring the potential effect of paricalcitol on markers of inflammation in de novo renal transplant recipients
**Short Title:**	Effect of paricalcitol on inflammatory pathways in renal transplantation
**Corresponding Author:**	Hege Kampen Pihlstrøm, PhD<br>Oslo Universitetssykehus<br>Oslo, NORWAY
**Keywords:**	Vitamin-D receptor agonist; paricalcitol; inflammation; fibrosis; osteoprotegerin; renal transplant recipients; gene expression analysis
**Abstract:**	Following a successful renal transplantation circulating markers of inflammation may remain elevated, and systemic inflammation is associated with worse clinical outcome in renal transplant recipients (RTRs). Vitamin D-receptor (VDR) activation is postulated to modulate inflammation and endothelial function. We aimed to explore if a synthetic vitamin D, paricalcitol, could influence systemic inflammation and immune activation in RTRs.<br><br>Newly transplanted RTRs were included in an open-label randomized controlled trial on the effect of paricalcitol on top of standard care over the first post-transplant year. Fifteen pre-defined circulating biomarkers reflecting leukocyte activation, endothelial activation, fibrosis and general inflammatory burden were analyzed in 74 RTRs at 8 weeks (baseline) and 1 year post-engraftment. Mean changes in plasma biomarker concentrations were compared by t-test. The expression of genes coding for the same biomarkers were investigated in 1-year surveillance graft biopsies (n=60).<br><br>In patients treated with paricalcitol circulating osteoprotegerin levels increased by 0.19 ng/ml, compared with a 0.05 ng/ml increase in controls (p=0.030). In graft tissue, a 21% higher median gene expression level of TNFRSF11B coding for osteoprotegerin was found in paricalcitol-treated patients compared with controls (p=0.026). Paricalcitol treatment did not significantly affect the blood- or tissue levels of any other investigated inflammatory marker.<br><br>In RTRs, paricalcitol treatment might increase both circulating and tissue levels of osteoprotegerin, a modulator of calcification, but potential anti-inflammatory treatment effects in RTRs are likely very modest.
**Order of Authors:**	Hege Kampen Pihlstrøm, PhD
	Thor Ueland
	Annika E Michelsen
	Pål Aukrust
	Franscesca Gatti
	Clara Hammarström
	Monika Kasprzycka
	Junbai Wang
	Guttorm Haraldsen
	Geir Mjøen
	Dag Olav Dahle

Powered by Editorial Manager® and ProduXion Manager® from Aries Systems Corporation

	Karsten Midtvedt
	Ivar Anders Eide
	My Hanna Sofia Svensson
	Anders Hartmann
	Hallvard Holdaas
**Additional Information:**	
**Question**	**Response**
**Financial Disclosure**<br><br>Enter a financial disclosure statement that describes the sources of funding for the work included in this submission. Review the submission guidelines for detailed requirements. View published research articles from *PLOS ONE* for specific examples.<br><br>This statement is required for submission and **will appear in the published article** if the submission is accepted. Please make sure it is accurate.<br><br>**Unfunded studies**<br>Enter: *The author(s) received no specific funding for this work.*<br><br>**Funded studies**<br>Enter a statement with the following details:<br>• Initials of the authors who received each award<br>• Grant numbers awarded to each author<br>• The full name of each funder<br>• URL of each funder website<br>• Did the sponsors or funders play any role in the study design, data collection and analysis, decision to publish, or preparation of the manuscript?<br>• **NO** - Include this sentence at the end of your statement: *The funders had no role in study design, data collection and analysis, decision to publish, or preparation of the manuscript.*<br>• **YES** - Specify the role(s) played.<br><br>* typeset	The trial was funded via a PhD grant to Hege K Pihlstrøm from South Eastern Norway Regional Health Authority (https://www.helse-sorost.no) and received an additional research grant from the Norwegian Society of Nephrology (https://nephro.no). Norwegian Health Authorities (https://helfo.no) also funded the trial by covering all expenses associated with paricalcitol treatment.The funders had no role in study design, data collection and analysis, decision to publish, or preparation of the manuscript.
**Competing Interests**<br><br>Use the instructions below to enter a competing interest statement for this submission. On behalf of all authors,	The authors have declared that no competing interests exist.

disclose any competing interests that could be perceived to bias this work—acknowledging all financial support and any other relevant financial or non-financial competing interests.<br><br>This statement **will appear in the published article** if the submission is accepted. Please make sure it is accurate. View published research articles from *PLOS ONE* for specific examples.<br><br>**NO authors have competing interests**<br><br>Enter: *The authors have declared that no competing interests exist.*<br><br>**Authors with competing interests**<br><br>Enter competing interest details beginning with this statement:<br><br>*I have read the journal's policy and the authors of this manuscript have the following competing interests: [insert competing interests here]*<br><br>* typeset	
**Ethics Statement**<br><br>Enter an ethics statement for this submission. This statement is required if the study involved:<br><br>• Human participants<br>• Human specimens or tissue<br>• Vertebrate animals or cephalopods<br>• Vertebrate embryos or tissues<br>• Field research<br><br>Write "N/A" if the submission does not require an ethics statement.<br><br>General guidance is provided below. Consult the submission guidelines for detailed instructions. **Make sure that all information entered here is included in the Methods section of the manuscript.**	The study was approved by the Regional Ethics Committee (REK no 2012/107) and the hospital´s Research Administration. It was also approved by the The Norwegian Medicines Agency, SLV (Eudract no: 2012-000429-32).<br>All participants signed written informed consent.

Powered by Editorial Manager® and ProduXion Manager® from Aries Systems Corporation

**Format for specific study types**<br><br>**Human Subject Research (involving human participants and/or tissue)**<br>• Give the name of the institutional review board or ethics committee that approved the study<br>• Include the approval number and/or a statement indicating approval of this research<br>• Indicate the form of consent obtained (written/oral) or the reason that consent was not obtained (e.g. the data were analyzed anonymously)<br><br>**Animal Research (involving vertebrate animals, embryos or tissues)**<br>• Provide the name of the Institutional Animal Care and Use Committee (IACUC) or other relevant ethics board that reviewed the study protocol, and indicate whether they approved this research or granted a formal waiver of ethical approval<br>• Include an approval number if one was obtained<br>• If the study involved *non-human primates*, add *additional details* about animal welfare and steps taken to ameliorate suffering<br>• If anesthesia, euthanasia, or any kind of animal sacrifice is part of the study, include briefly which substances and/or methods were applied<br><br>**Field Research**<br><br>Include the following details if this study involves the collection of plant, animal, or other materials from a natural setting:<br>• Field permit number<br>• Name of the institution or relevant body that granted permission	
**Data Availability**<br><br>Authors are required to make all data underlying the findings described fully available, without restriction, and from the time of publication. PLOS allows rare exceptions to address legal and ethical concerns. See the PLOS Data Policy and FAQ for detailed information.	No - some restrictions will apply

A Data Availability Statement describing where the data can be found is required at submission. Your answers to this question constitute the Data Availability Statement and **will be published in the article**, if accepted.<br><br>**Important:** Stating 'data available on request from the author' is not sufficient. If your data are only available upon request, select 'No' for the first question and explain your exceptional situation in the text box.<br><br>Do the authors confirm that all data underlying the findings described in their manuscript are fully available without restriction?	
**Describe where the data may be found in full sentences. If you are copying our sample text, replace any instances of XXX with the appropriate details.**<br><br>• If the data are **held or will be held in a public repository**, include URLs, accession numbers or DOIs. If this information will only be available after acceptance, indicate this by ticking the box below. For example: *All XXX files are available from the XXX database (accession number(s) XXX, XXX.).*<br>• If the data are all contained **within the manuscript and/or Supporting Information files**, enter the following: *All relevant data are within the manuscript and its Supporting Information files.*<br>• If neither of these applies but you are able to provide **details of access elsewhere**, with or without limitations, please do so. For example:<br><br>*Data cannot be shared publicly because of [XXX]. Data are available from the XXX Institutional Data Access / Ethics Committee (contact via XXX) for researchers who meet the criteria for access to confidential data.*<br><br>*The data underlying the results presented in the study are available*	Norwegian Data Protection Authorities have a strict practice concerning access to research data. Access to data collected from this study, including de-identified individual participant data, will be made available following publication upon email request to the corresponding author. Data will be shared with investigators whose proposed use of the data has been approved by the project steering committee and are according to the consent given by the participants and Norwegian laws and legislations. The trial protocol is available as a supplement to this manuscript.<br><br>However, fully anonymized microarray data have been made accessible through the GEO database (http://www.ncbi.nlm.nih.gov/geo/query/acc.cgi?acc=GSE83486).

Powered by Editorial Manager® and ProduXion Manager® from Aries Systems Corporation

from (include the name of the third party and contact information or URL).<br>• This text is appropriate if the data are owned by a third party and authors do not have permission to share the data.<br><br>* typeset	
Additional data availability information:	

Manuscript
Click here to access/download;Manuscript;Paricalcitol inflammation 160620.docx

Exploring the potential effect of paricalcitol on markers of inflammation in *de novo* renal transplant recipients

Hege Kampen Pihlstrøm[1], Thor Ueland[2,3,4], Annika E Michelsen[2,3], Pål Aukrust[2,3,5], Franscesca Gatti [6,7], Clara Hammarström[6,7], Monika Kasprzycka[6,7], Junbai Wang[6], Guttorm Haraldsen[6,7], Geir Mjøen[1], Dag Olav Dahle[1], Karsten Midtvedt[1], Ivar Anders Eide[8],My Hanna Sofia Svensson[3,8],Anders Hartmann[1,3], Hallvard Holdaas[1]

[1] Department of Surgery, Inflammation Medicine and Transplantation, Section of Nephrology, Oslo University Hospital, Rikshospitalet, Oslo, Norway.

[2]Research Institute of Internal Medicine, Oslo University Hospital Rikshospitalet, Oslo, Norway

[3]Institute of Clinical Medicine, Faculty of Medicine, University of Oslo, Oslo, Norway

[4]K.G. Jebsen Thrombosis Research and Expertise Center , University of Tromsø, Tromsø, Norway

[5] Department of Surgery, Inflammation Medicine and Transplantation, Section of Clinical Immunology and Infectious Diseases, Oslo University Hospital, Rikshospitalet, Oslo, Norway.

[6]Department of Pathology, Oslo University Hospital, Oslo, Norway

[7]K.G.Jebsen Inflammation Research Centre, Laboratory of Immunohistochemistry and Immunopathology, University of Oslo, Oslo, Norway

[8]Department of Nephrology, Division of Medicine, Akershus University Hospital, Oslo, Norway.

Corresponding author:

Hege Kampen Pihlstrøm,

E-mail: hegphi@ous-hf.no.

Clinical Trial Notation

The study has been registered as NCT01694160 (2012/107D) on www.clinicaltrials.gov.

Abbreviations

CI	confidence interval
CKD	chronic kidney disease
CNI	calcineurin inhibitor
CRP	C-reactive protein
DLL1	delta like canonical Notch ligand 1
ECM	extracellular matrix
ELISA	enzyme-linked immunosorbent assay
eGFR	estimated glomerular filtration rate
hsCRP	high-sensitive C-reactive protein
IL-6	interleukine-6
MMP-9	matrix metalloprotease-9
mRNA	messenger ribonucleic acid
NGAL	neutrophil gelatinase-associated lipocalin
OPG	osteoprotegerin
PAT	peripheral arterial tonometry
PTH	parathyroid hormone
RAAS	renin-angiotensin-aldosterone system
RANK	Receptor activator of nuclear factor kappa-B
RANKL	Receptor activator of nuclear factor kappa-B ligand
RTRs	renal transplant recipients
SD	standard deviation
sIL2Ra	soluble interleukin-2 receptor alpha
TGF-β	transforming growth factor beta
TIMP-1	tissue inhibitor of metalloproteinase -1
TNF	tumor necrosis factor
vWF	von Willebrand factor
VDRA	vitamin D receptor agonist

Abstract

Following ~~a~~ successful renal transplantation circulating markers of inflammation may remain elevated, and systemic inflammation is associated with worse clinical outcome ~~in renal transplant recipients (RTRs)~~. Vitamin D-receptor (VDR) activation is postulated to modulate inflammation and endothelial function. We aimed to explore if a synthetic vitamin D, paricalcitol, could influence systemic inflammation and immune activation in RTRs.

Newly transplanted RTRs were included in an open-label randomized controlled trial on the effect of paricalcitol on top of standard care over the first post-transplant year. Fifteen pre-defined circulating biomarkers reflecting leukocyte activation, endothelial activation, fibrosis and general inflammatory burden were analyzed in 74 RTRs at 8 weeks (baseline) and 1 year post-engraftment. Mean changes in plasma biomarker concentrations were compared by *t*-test. The expression of genes coding for the same biomarkers were investigated in 1-year surveillance graft biopsies (n=60).

In patients treated with paricalcitol circulating osteoprotegerin levels increased by 0.19 ng/ml, compared with a 0.05 ng/ml increase in controls (p=0.030). In graft tissue, a 21% higher median gene expression level of TNFRSF11B coding for osteoprotegerin was found in paricalcitol-treated patients compared with controls (p=0.026). Paricalcitol treatment did not significantly affect the blood- or tissue levels of any other investigated inflammatory marker.

In RTRs, paricalcitol treatment might increase both circulating and tissue levels of osteoprotegerin, a modulator of calcification, but potential anti-inflammatory treatment effects in RTRs are likely very modest.

(ClinicalTrials.gov number NCT01694160 (2012/107D))

Introduction

Chronic kidney disease (CKD) is associated with systemic inflammation. In end-stage renal disease there is a strong association between inflammation marker C-reactive protein (CRP) and risk of death and cardiovascular events [1]. Increased oxidative stress and chronic activation of various cell subsets of both the innate and adaptive immune system creates a pro-inflammatory milieu in CKD [2]. Following a successful renal transplantation with appropriate immunosuppression obtained by a

this is very vague terminology

combination of drugs, the inflammatory imbalance in the immune system [3, 4] is ameliorated. Some derangements in immune function will however persist [5, 6], especially if graft function is sub-optimal. Even among renal transplant recipients (RTRs) with low Framingham risk scores, implying a limited burden of comorbidity, there is evidence for enhanced systemic inflammation [7]. In stable

No, Il-6 can control hepatic CRP generation

RTRs, interleukin-6 (IL-6) and its major product, CRP, has been associated with risk of major cardiovascular events and all-cause mortality [8], as has neopterin [9, 10], a marker of interferon (IFN) γ-mediated activation of monocytes/macrophages.

Vitamin D is a fat-soluble vitamin central to the maintenance of bone- and mineral homeostasis. Poor vitamin D status has also been associated with increased risk of cancer[11], infections[12, 13], autoimmune diseases[14], cardiovascular events[15], obesity[16] and diabetes[17]. However, interventional studies on vitamin D supplementation report equivocal and inconsistent effects on non-skeletal clinical outcomes, and larger randomized controlled trials are ongoing [18-20]. Anti-inflammatory and immune-modulating effects of vitamin D -supplements could be of particular benefit for renal transplant patients, as they are prone to vitamin D-deficiency, while at the same time carrying an increased risk of all of the above mentioned chronic conditions [21, 22].

Paricalcitol (19-nor-1,25-dihydroxyvitamin D2), a synthetic selective third generation vitamin D-receptor agonist (VDRA), is used in patients with CKD to treat secondary hyperparathyroidism. Potential non-skeletal benefits of paricalcitol include anti-proteinuric effects in patients with

diabetes[23] and in RTRs[24]. VDRAs seem also to have anti-inflammatory potential, and experimental studies have indicated dampened tumor necrosis factor (TNF) and interleukine-8 (IL-8) production[25] and reduced inflammation and fibrosis development [26] .

An observational study in RTRs with secondary hyperparathyroidism found that 3 months of treatment with paricalcitol reduced serum IL-6 and TNF levels, with corresponding lowered mRNA expression in peripheral blood mononuclear cells [27]. In a clinical trial of 168 RTRs with proteinuria, paricalcitol treatment on top of RAAS-blockade caused significant reductions in circulating IL-6 and the pro-fibrotic mediator transforming growth factor beta (TGF-β)[28]. We were, however, not able to demonstrate a treatment effect of paricalcitol on high sensitity (hs) CRP in 77 newly transplanted RTRs [29], but it is unlikely that hsCRP reflects all inflammatory pathways that are activated in RTRs.

Cytokines and inflammatory molecules are operating in a complex network, and our aim in the current study on renal transplant recipients was to explore the potential effect of paricalcitol on a broader range of pre-defined circulating inflammatory markers, including markers reflecting leukocyte activation, endothelial activation, fibrosis and more general vascular inflammation.

Materials and methods

Study design

From Jan 2013 up until Jan 2014, 77 patients >18 years of age who had received a kidney transplant or a combined kidney-pancreas transplant were randomized to receive either treatment with paricalcitol 2 μg daily or standard post-transplant care. Study randomization and baseline laboratory measurements took place 7-8 weeks after transplantation. The last study visit was scheduled one year after date of engraftment and included repeated blood sampling and collection of renal transplant biopsy tissue for subsequent histopathology evaluation and RNA extraction. To be eligible for the study, patients should have an estimated glomerular filtration rate (eGFR) by the CKD-EPI formula of at least 30 ml/min, total serum calcium levels should range between 2.0 and 2.6 mmol/l, and the immunosuppressive regimen should include a calcineurin inhibitor (CNI). Patients already undergoing treatment with vitamin D, VDRA or calcimimetic drugs were excluded, as were patients with established osteoporosis in the axial skeleton, patient with a history of hypersensitivity towards paricalcitol or related drugs and recipients receiving organs from a donor older than 75 years. Results concerning the primary endpoint (potential anti-proteinuric effect) and main secondary endpoints are found elsewhere [29], as is the Consort diagram presenting the screening- and inclusion process. All patients provided written informed consent before inclusion in the trial. The study conformed to the principles of the Declaration of Helsinki and the Declaration of Istanbul, and the protocol was approved by the Regional Ethics Committee and the hospital´s Research Administration. The Department of Organ Transplantation at Oslo University Hospital was responsible for the coordination and conduction of the trial.

Outcomes

Cytokines and inflammatory molecules are operating in a complex network, and our aim in the current study was to explore the potential effect of paricalcitol on a broader range of stable and readily measurable circulating inflammatory markers. We included biomarkers reflecting *leukocyte activation* (soluble IL-2 receptor alpha [sIL2Ra] as a marker of T-cell activation; neopterin, soluble CD14 and soluble CD163 as markers of monocyte/macrophage activation; neutrophil gelatinase-associated lipocalin [NGAL] as a marker of neutrophil activation), *endothelial activation* (von Willebrand factor [vWf], and angiopoietin-2), *fibrosis* (endostatin, matrix metalloprotease-9 [MMP-9], galectin3, tissue inhibitor of metalloproteinase 1 [TIMP-1], activin A) and more *general vascular inflammation* (osteoprotegerin [OPG]), soluble tumor necrosis factor-receptor 1 [TNFR 1] and delta like canonical Notch ligand 1 [DLL1]). As a supplementary analysis the levels of mRNA reflecting expression of genes coding for the same biomarkers were investigated in renal graft tissue at study end.

Laboratory

After an overnight fast, blood samples were drawn in the morning at the baseline visit and at the last study visit one year after transplantation. After centrifugation at 2350 g for 10 minutes, plasma and serum were immediately frozen and samples stored at -72° C until analysis (April-May 2018). Neopterin levels were analyzed by enzyme-linked immunosorbent assay (ELISA) from IBL International GmbH (Hamburg, Germany), while the remaining markers were analyzed using ELISAs from RnD-systems (Minneapolis, Minnesota, USA).

Gene expression analyses

The procedure of RNA extraction from retrieved transplant biopsy tissue stored in RNA*later*® solution, together with RNA quality assessment, amplification- and labelling procedures are

described elsewhere [29]. Sixty samples, equally divided between study groups, were found to have sufficient quality for microarray gene expression analyses. For this post-hoc investigation, we selected 18 gene products reflecting the expression of 14 biomarker proteins. (Neopterin is a degradation product for which the circulating level is unlikely to be directly reflected by the expression any gene).

Statistical methods

The intention-to-treat population consisted of any patient who was randomized and, if assigned to the treatment group, received at least one dose of study drug, irrespective of any study protocol violation. The per-protocol population consisted of participants actually fulfilling the protocol requirements for eligibility, intervention and outcome assessment.

Comparisons of baseline variables between study groups were done using *t*-test, Mann-Whitney U Test or Pearson $\chi 2$ as found appropriate. The potential correlation between change in osteoprotegerin and change in PTH was tested using Pearsons Correlation test.

The levels of biomarkers in the circulation, expressed as *change from baseline to study end*, showed only marginal deviations from a normal distribution, rendering the *t*-test for independent observations applicable. As a sensitivity analysis, ANCOVA was performed, analyzing group differences in levels of biomarkers at study end with adjustments for baseline levels.

For analyses of microarray data, gene expression levels were log transformed, and normalized intensities were converted to Z-scores, which were used to identify differentially expressed genes between the paricalcitol group and controls. For each gene, a relative ratio of the mean Z-scores between the two groups was computed, and the statistical significance of relative ratios (P-value) was estimated by the two-sample Kolmogorov-Smirnov goodness-of-fit hypothesis test (KS-test), which does not have a prior assumption for the distribution of gene expression [30].

Statistical analyses of circulating biomarker levels were performed in SPSS version 21 (IBM, New York, USA). Microarray gene expression data analysis were performed by either MATLAB statistics toolbox (MathWorks, Natick, USA) or in-house script files such as Python based on previously published works[31].

Results

Baseline characteristics

A total of 74 patients had available plasma samples for analyses of circulating biomarkers at baseline and study end. Baseline demographics, as well as relevant laboratory values and vital signs are presented for both study groups in Table 1. There were no statistically significant differences between groups, but there was a trend towards more males (p=0.19) and a lower baseline vitamin 25-OH-D (p=0.16) in the control group. Twenty-eight patients (38%), equally divided between study groups, had an immunological cause of end-stage renal disease, e.g. glomerulonephritis.

Table 1. Baseline characteristics of the study population[a].

Variables	Paricalcitol (n=35)	Control (n=39)
Age, years	55.6 (13.3)	55.1 (12.6)
Male gender	26 (74%)	33 (85%)
Caucasian ethnicity	34 (97%)	37 (95%)
BMI, kg/m^2	26.2 (3.3)	25.5 (3.9)
Current smoking	5 (14%)	5 (13%)
Living donor	10 (29%)	12 (31%)
Cold ischemia time, hours	10.4 (6.4)	10.1 (5.7)
Glomerulonephritis as cause of CKD	13 (37.1)	15 (38.5)
Predialytic	13 (37%)	13 (33%)
Hypertension	29 (83%)	36 (92%)
Chronic heart disease	11 (31%)	13 (33%)
Pre-tx diabetes mellitus	6 (17%)	6 (15%)
Systolic blood pressure, mmHg	145 (21)	143 (22)
Diastolic blood pressure, mmHg	83 (10)	84 (11)
Treatment with ACEi/ARB, %	9 (26%)	14 (36%)
Cholesterol, mmol/L	5.8 (1.1)	5.9 (0.9)
HDL cholesterol, mmol/L	1.6 (0.5)	1.6 (0.4)
LDL cholesterol, mmol/L	3.8 (1.0)	3.9 (0.9)
Triglycerides, mmol/L *	1.3 (1.0)	1.4 (0.5)
Creatinine, µmol/L	115 (25)	122 (30)
Hemoglobin, g/L	12.4 (1.2)	12.3 (1.2)
hsCRP, mg/L ¤ *	0.85 (2.20)	1.00 (1.19)
Calcium total, mmol/L	2.38 (0.09)	2.34 (0.21)

Phosphate, mmol/L	*	0.9 (0.3)	0.8 (0.4)
Albumin, g/L		42.3 (2.5)	41.5 (2.4)
PTH, pmol/L	*	10.1 (9.2)	10.2 (5.4)
Alkaline phosphatase, U/L		60.7 (21.8)	69.4 (28.6)
Vitamin 25-OH-D, nmol/l		50.1 (18.0)	44.8 (17.2)
Urine Albumin/creatinine ratio, mg/mmol	*	3.1 (7.4)	4.5 (8.7)

BMI, body mass index; CKD, chronic kidney disease; HDL, high density lipoprotein; hsCRP, high-sensitive C-reactive protein; LDL, low density lipoprotein; PTH, parathyroid hormone; ACEi, angiotensin converting enzyme inhibitor; ARB, angiotensin receptor blocker.
All laboratory measurements are performed in plasma.
[a]Modified version of table from the original publication[29].
Continuous data expressed as mean (standard deviation) or * median (interquartile range).
Categorical data expressed as number (percentage frequency).
¤ Values <0.60mg/L (laboratory detection cut-off) are all given the value 0.30. Values >15mg/L are rounded down to this value.

Two patients were diagnosed with biopsy-proven antibody mediated rejection at 1-year follow up, while four patients were being treated for acute cellular rejection at time of study inclusion or earlier postoperatively. Two patients suffered a cellular rejection in the interval between study visits. Seven patients suffered one or more infections needing systemic antibiotic treatment over the duration of the study. None of these participants have been excluded from the primary efficacy analyses.

Effects of paricalcitol on serum levels of inflammatory markers

Paricalcitol did not significantly reduce the levels of measured inflammatory markers as compared with no treatment. Table 2 presents intention-to-treat analyses, showing the mean (or median) levels of inflammatory biomarkers in each group at 8 weeks and 1 year post transplant, together with the changes from baseline to study end given in percent of baseline mean (or median). *T*-test for likelihood of observing the reported results given no true group difference is presented with corresponding p-values and confidence interval for the absolute group difference in change. MMP-9 levels decreased in the paricalcitol group, while it increased in the control group, but the difference was not statistically significant (difference in change [ng/ml], p=0.058; CI -56.3 - 0.95). Paricalcitol treatment was, however, associated with *increased* mean OPG levels, as opposed to nearly no change in the control group (difference in change [ng/ml], p=0.030; CI 0.01-0.26). A few extreme

values were responsible for at least some of this difference (Fig 1). For all other parameters, p-values for differences in changes were >0.1 and potential effect sizes were small (Table 2).

Fig 1. Changes in levels of osteoprotegerin across the study period.

Osteoprotegerin change (ng/nl) in patients treated with paricalcitol vs patients receiving no extra treatment; median (horizontal line), interquartile range (blue box), outlier (°).

Table 2. Plasma levels of biomarkers at baseline and study end, by treatment group.

Biomarker (plasma levels)	Paricalcitol (n=35)			Control (n=39)			*t*-test
	Baseline Mean (SD)	1-year Mean (SD)	Change (%)	Baseline Mean (SD)	1 year Mean (SD)	Change (%)	p-values (CI ng/ml) for group differences in change
Angiopoietin-2 (ng/ml)*	0.74 (0.49)	0.69 (0.41)	- 6.8	0.67 (0.46)	0.72 (0.55)	+ 7.5	0.478 (-0.57 – 0.27)
sCD14 (ng/ml)	1.57 (0.21)	1.57 (0.30)	+ 0.0	1.58 (0.27)	1.52 (0.29)	- 3.8	0.383 (-0.08 – 0.21)
sCD163 (ng/ml)	441 (230)	547 (316)	+ 24.0	467 (238)	512 (190)	+ 9.6	0.241 (-42.3 – 165.6)
DLL1 (ng/ml)	8.88 (2.07)	9.73 (2.83)	+ 9.6	9.62 (2.40)	9.85 (2.84)	+ 2.4	0.212 (-0.37 – 1.62)
Endostatin (ng/ml)	99.1 (21.8)	93.4 (21.6)	- 5.8	108.7 (32.2)	103.0 (32.1)	- 5.2	0.908 (-9.94 – 11.17)
sIL2ra (pg/ml)	3896 (1971)	369 (402)	- 90.5	3202 (1877)	336 (303)	- 89.5	0.152 (-1572 – 249)
MMP9 (ng/ml)	99.8 (67.7)	74.4 (43.7)	- 25.4	77.4 (35.9)	79.7 (78.8)	+ 2.3	0.058 (-56.3 – 0.95)
sTNFr1(ng/ml)	1.95 (0.67)	2.01 (0.91)	+ 3.1	2.22 (0.78)	2.16 (0.79)	- 2.7	0.464 (-0.21 – 0.46)
Galectin-3 (ng/ml)	1.41 (0.56)	1.17 (0.55)	- 17.1	1.48 (0.57)	1.26 (0.44)	- 14.9	0.835 (-0.25 – 0.20)
NGAL (ng/ml)	282 (122)	312 (144)	+ 10.6	287 (145)	305 (133)	+ 6.5	0.735 (-52.0 – 73.3)
vWF in % of ref.plasma*	82.5 (89.5)	57.1(37.0)	- 30.8	102.8 (118.3)	57.8 (58.6)	- 43.8	0.228 (-15.1 – 62.3)
ActivinA (ng/ml)*	344 (739)	323 (1075)	- 6.1	578 (910)	681 (873)	+ 17.8	0.696 (-206 – 306)
OPG (ng/ml)	0.91 (0.37)	1.10 (0.44)	+ 20.9	1.08 (0.53)	1.13 (0.52)	+ 4.6	0.030 (0.01 – 0.26)
TIMP-1 (ng/ml)	110 (23)	112 (26)	+ 1.8	124 (32)	122 (38)	- 1.6	0.461 (-7.17 – 15.6)
Neopterin (nmol/L)*	21.4 (18.5)	21.1 (13.8)	+ 1.4	22.1 (13.7)	20.4 (11.4)	+ 7.7	0.787 (-12.5 – 16.3)

Intention-to-treat population. *T*-test for difference in change: p-values presented with corresponding confidence intervals (CI).

DLL1, delta like canonical Notch ligand 1; sIL2Ra, soluble interleukin-2 receptor alpha; MMP9, matrix metalloprotease-9; sTNFR1, soluble tumor necrosis factor receptor-1; NGAL, neutrophil gelatinase-associated lipocalin ; vWF, von Willebrand factor; OPG, osteoprotegerin; TIMP-1, Tissue inhibitor of metalloproteinase 1

No significant correlation was found between change in osteoprotegerin and change in PTH (p=0.74). Sensitivity analyses using ANCOVA did not materially change results: p= 0.062 for difference between groups in OPG at 1-year adjusted for baseline OPG level (data not shown).

Analyses of the per-protocol population (n=67) did not change results (p=0.041 for increase in OPG and p=0.076 for reductions in MMP-9 with paricalcitol treatment). Results of sensitivity analyses excluding patients diagnosed with rejection at any time point during follow up (n=67) were also comparable to the main analysis (p=0.023 for increase in OPG and p=0.071 for reductions in MMP-9). Exclusion of patients with glomerulonephritis as cause of ESRD did not affect results (n=45, OPG; p=0.057, MMP-9; p=0.018).

Gene expression in renal graft tissue in response to paricalcitol

In renal graft tissue of patients treated with paricalcitol, there was a 21% higher expression of TNFRSF11B , the gene coding for osteoprotegerin , compared with the control group (median gene expression 0.808 vs 0.668; p=0.026). We detected no other significant differences in the expression of biomarker genes between patients treated with paricalcitol and controls, as illustrated by the heat map (Fig 2). All microarray data are available at the Gene Expression Omnibus (GEO) database; http://www.ncbi.nlm.nih.gov/geo/query/acc.cgi?acc=GSE83486.

Fig 2. Heat map of inflammatory marker gene expression levels in graft tissue.

The expression of 18 genes coding for 14 inflammatory biomarkers in 30 treated patients (to the left) vs 30 patients in the control group (to the right). Darker color indicates higher expression levels. Z-scores of duplicated genes in the array are averaged.

Discussion

We explored the potential effects of paricalcitol treatment during the first year after kidney transplantation on a wide range of biomarkers reflecting several aspects of inflammatory responses, but were unable to confirm clinically or statistically significant effects ameliorating inflammation. Paricalcitol treatment did, however, increase circulating OPG levels. Importantly, this was accompanied by a corresponding increase in TNFRSF11b, the gene coding for OPG, in biopsies from renal allografts, supporting a link between paricalcitol and OPG.

The proposed anti-inflammatory effects of paricalcitol are ascribed to a regulatory role of vitamin D receptor activation in several subsets of immune cells, such as macrophages, dendritic cells and T-cells[32, 33]. However, in one clinical study in patients with vascular inflammation, paricalcitol appeared to exert its effect rather selectively on T-helper cells by interfering with calcineurin-mediated responses [34]. Modulation of adaptive immune responses in CKD-patients has been demonstrated, reflected by reductions in several inflammatory markers of Th1- Th2 and Th17-responses[35]. Oblak et al[28] demonstrated treatment effects in a ~~quite~~ large interventional study of RTRs. However, most clinical trials suggesting anti-inflammatory properties of paricalcitol in CKD-patients [36-39] and RTRs [40] has been hampered by a limited sample size. The adequately powered VITAL-study (n=281) failed to show significant effects of paricalcitol on a range of inflammatory biomarkers in type 2 diabetes mellitus[23]. Taken together, the results of interventional studies on vitamin D agonist treatment seem inconclusive.

In the present study we found no evidence of paricalcitol influencing markers reflecting monocyte/macrophage (i.e. sCD163, sCD14, neopterin), T cell (i.e. sCD25) or neutrophil (i.e. NGAL) activation, which signals no major clinically beneficial effect of paricalcitol on the activation and interplay of immune cells in the context of kidney transplantation. However, in light of recent findings suggesting that paricalcitol modulates inflammatory responses by influencing the

calcineurin-axis [34], is it possible that anti-inflammatory effects could be masked by calcineurin inhibitor treatment, the corner stone in the immunosuppressive regimen for all our study participants.

Paricalcitol appears also to reduce development of renal interstitial fibrosis in obstructive nephropathy [41] and RTRs [42]. Metalloproteases, including MMP-9, are major regulators of ECM protein metabolism [43].. One might be tempted to interpret the trend towards reduced MMP-9 in paricalcitol-treated patients as a potential effect of VDRA on tissue extracellular matrix (ECM) remodeling, but it remains a speculation. A major source of MMP-9 in the circulation is neutrophils [44], hence plasma levels might also to some extent reflect neutrophil activation status.

We found that patients randomized to paricalcitol experienced an *increase* in circulating levels of OPG not seen in the control group. Correspondingly, the expression of the gene TNFRSF11B coding for OPG was also higher in renal graft tissue of patients in the intervention group. The result is consistent with experimental data on the immunomodulatory effects of 1,25-hydroxyvitamin D3[45] and a similar clinical trial in hemodialysis patients[46]. Hansen *et al* [46] found the rise in OPG in patients treated with paricalcitol to be correlated with the degree of suppression of PTH, partly explaining their results. Such a correlation was not clear in our cohort, despite a significant PTH-lowering effect of paricalcitol[29]. OPG protects the skeleton from excessive bone resorption by attaching to receptor activator of nuclear factor kappa-B ligand (RANKL) and preventing it from binding to its receptor on osteoclasts, RANK[47]. Plasma OPG has been suggested as a stable marker of the general activity in the RANKL/RANK system, a system that is linked to fibrogenesis and regulation of extracellular matrix. It is debated whether OPG itself is cardioprotective or a reactive proinflammatory molecule [48, 49]but modulatory roles in vascular injury and calcification, systemic inflammation and atherosclerosis, as well as in fibrosis pathways have been suggested [50, 51]. Thus, together with the potential downregulation of MMP-9 the effect seen on OPG may suggest that paricalcitol could have some effect on fibrogenesis in RTRs.

Interpretation of findings

Since inflammatory markers typically have a wide distribution and relatively large SD's, the power to detect group differences in a moderate-sized study, such as ours, could be lower than anticipated. As an example, MMP-9-levels were reduced by mean 25 ng/ml in the intervention group, while the controls had a 2 ng/ml rise during the study period. However, a 45 ng/ml difference in treatment effect would be needed to claim statistical significance. This is a high threshold for a biomarker whose reference range in healthy males is approximately 20 – 100 (M ±2SD) ng/ml[52].

Effect sizes may be more relevant that any p-value in itself[53]. Thus, although significant p-values are lacking in the current study, our results should not be interpreted as firm evidence against a potential anti-inflammatory effect of paricalcitol. Instead our results signal possible small-to-moderate effects within the limits of the reported confidence intervals. Taken together, evidence nevertheless seems too inconsistent to motivate the routine use of VDRA to reduce inflammation or improve vascular health in the transplant population. However, the interaction between paricalcitol and OPG as seen both at protein and transcript levels should be further explored as a potential important target for VDRAs.

Strengths and limitations

There was a high level of adherence to treatment in the paricalcitol group and no patient-initiated withdrawals [29]. The study population has been well characterized. Both circulating biomarker levels and tissue biomarker gene expression were evaluated, thus increasing the robustness of the results. However, sample size was calculated for the primary trial endpoint, not for the detection of potential treatment effects on levels of inflammatory markers. Notably, this study is of an explorative nature, testing many biomarkers at the same time. The suggested association between VDRA and OPG must be interpreted with caution, due to the possibility of making type 1-errors when performing multiple statistical tests. Conclusions drawn from this study might only be valid for a

white European population of RTRs with a reasonably good allograft function (i.e. eGFR>30 ml/min). Results are not necessarily applicable for recipients with vitamin D deficiency. We acknowledge that investigational bias might be a problem in open label trials, but for administrative reasons placebo drugs were unfortunately not available.

Conclusions

In newly transplanted RTRs with adequate graft function, we were not able to demonstrate convincing reductions in levels of circulating biomarkers of inflammation and endothelial function after ten months of paricalcitol treatment. If present, a modulating effect of VDRA-treatment on systemic inflammation in this patient group is likely to be modest. We found that VDRA-treatment might increase levels of OPG, both in the circulation and in renal tissue, but this result needs to be replicated and validated.

Acknowledgements

This trial was funded via a PhD grant from South Eastern Norway Regional Health Authority (Helse Sør-Øst) and received an additional research grant from the Norwegian Society of Nephrology. The Norwegian Health Authority also provided the paricalcitol capsules.

Thanks to study participants, study nurses and bioengineers at the Kidney Physiology Research Laboratory.

References

1. Yeun JY, Levine RA, Mantadilok V, Kaysen GA. C-Reactive protein predicts all-cause and cardiovascular mortality in hemodialysis patients. Am J Kidney Dis. 2000;35(3):469-76. PubMed PMID: 10692273.
2. Vaziri ND. Oxidative stress in uremia: nature, mechanisms, and potential consequences. Semin Nephrol. 2004;24(5):469-73. PubMed PMID: 15490413.
3. Tabriziani H, Lipkowitz MS, Vuong N. Chronic kidney disease, kidney transplantation and oxidative stress: a new look to successful kidney transplantation. Clin Kidney J. 2018;11(1):130-5. doi: 10.1093/ckj/sfx091. PubMed PMID: 29423212; PubMed Central PMCID: PMCPMC5798135.
4. Gennip A, Broers NJH, Meulen KJT, Canaud B, Christiaans MHL, Cornelis T, et al. Endothelial dysfunction and low-grade inflammation in the transition to renal replacement therapy. PLoS One. 2019;14(9):e0222547. doi: 10.1371/journal.pone.0222547. PubMed PMID: 31518378; PubMed Central PMCID: PMCPMC6743867 Fresenius Medical Care Deutschland GmbH (study NL33129.068.10 and NL43381.068.13), a Baxter International extramural grant (study NL35039.068.10; grant reference number 11CECHHDEU1004) and the Dutch Kidney Foundation (study NL35039.068.10; grant reference number SB166). BC and AG, who are employees of Fresenius Medical Care and hold shares in the company, reviewed the manuscript. However, the funding sources had no role in the preparation or analysis of data, and the decision to publish. In addition, RJHM, NJHB and JPK are supported by an (un)restricted grant from Fresenius Medical Care. RJHM and NJHB received lectures fees from Fresenius Medical Care. MHC reports Astellas Travel support grants to visit the international conferences ESOT 2017 and TTS 2018. This does not alter our adherence to PLOS ONE policies on sharing data and materials.
5. Canas L, Iglesias E, Pastor MC, Barallat J, Juega J, Bancu I, et al. Inflammation and oxidation: do they improve after kidney transplantation? Relationship with mortality after transplantation. Int Urol Nephrol. 2017;49(3):533-40. doi: 10.1007/s11255-016-1435-4. PubMed PMID: 28013471.
6. van Riemsdijk-van Overbeeke IC, Baan CC, Loonen EH, Hesse CJ, Zietse R, van Gelder T, et al. The TNF-alpha system after successful living-related kidney transplantation. Transpl Int. 1998;11 Suppl 1:S46-9. doi: 10.1007/s001470050424. PubMed PMID: 9664942.
7. Mansell H, Rosaasen N, Dean J, Shoker A. Evidence of enhanced systemic inflammation in stable kidney transplant recipients with low Framingham risk scores. Clin Transplant. 2013;27(4):E391-9. doi: 10.1111/ctr.12159. PubMed PMID: 23782452.
8. Abedini S, Holme I, Marz W, Weihrauch G, Fellstrom B, Jardine A, et al. Inflammation in renal transplantation. Clin J Am Soc Nephrol. 2009;4(7):1246-54. doi: 10.2215/CJN.00930209. PubMed PMID: 19541816; PubMed Central PMCID: PMCPMC2709521.
9. Pihlstrom H, Mjoen G, Marz W, Olav Dahle D, Abedini S, Holme I, et al. Neopterin is associated with cardiovascular events and all-cause mortality in renal transplant patients. Clin Transplant. 2014;28(1):111-9. doi: 10.1111/ctr.12285. PubMed PMID: 24372612; PubMed Central PMCID: PMCPMC4204514.
10. Grebe SO, Kuhlmann U, Fogl D, Luyckx VA, Mueller TF. Macrophage activation is associated with poorer long-term outcomes in renal transplant patients. Clin Transplant. 2011;25(5):744-54. PubMed PMID: 20964718.
11. Tagliabue E, Raimondi S, Gandini S. Vitamin D, Cancer Risk, and Mortality. Adv Food Nutr Res. 2015;75:1-52. doi: 10.1016/bs.afnr.2015.06.003. PubMed PMID: 26319903.
12. Kalluri HV, Sacha LM, Ingemi AI, Shullo MA, Johnson HJ, Sood P, et al. Low vitamin D exposure is associated with higher risk of infection in renal transplant recipients. Clin Transplant. 2017;31(5). doi: 10.1111/ctr.12955. PubMed PMID: 28295622.
13. Watkins RR, Lemonovich TL, Salata RA. An update on the association of vitamin D deficiency with common infectious diseases. Can J Physiol Pharmacol. 2015;93(5):363-8. doi: 10.1139/cjpp-2014-0352. PubMed PMID: 25741906.

14. Ishikawa LLW, Colavite PM, Fraga-Silva TFC, Mimura LAN, Franca TGD, Zorzella-Pezavento SFG, et al. Vitamin D Deficiency and Rheumatoid Arthritis. Clin Rev Allergy Immunol. 2017;52(3):373-88. doi: 10.1007/s12016-016-8577-0. PubMed PMID: 27484684.
15. Muscogiuri G, Annweiler C, Duval G, Karras S, Tirabassi G, Salvio G, et al. Vitamin D and cardiovascular disease: From atherosclerosis to myocardial infarction and stroke. Int J Cardiol. 2017;230:577-84. doi: 10.1016/j.ijcard.2016.12.053. PubMed PMID: 28043680.
16. Walsh JS, Bowles S, Evans AL. Vitamin D in obesity. Curr Opin Endocrinol Diabetes Obes. 2017;24(6):389-94. doi: 10.1097/MED.0000000000000371. PubMed PMID: 28915134.
17. Berridge MJ. Vitamin D deficiency and diabetes. Biochem J. 2017;474(8):1321-32. doi: 10.1042/BCJ20170042. PubMed PMID: 28341729.
18. Manson JE, Bassuk SS, Lee IM, Cook NR, Albert MA, Gordon D, et al. The VITamin D and OmegA-3 TriaL (VITAL): rationale and design of a large randomized controlled trial of vitamin D and marine omega-3 fatty acid supplements for the primary prevention of cancer and cardiovascular disease. Contemp Clin Trials. 2012;33(1):159-71. doi: 10.1016/j.cct.2011.09.009. PubMed PMID: 21986389; PubMed Central PMCID: PMCPMC3253961.
19. Neale RE, Armstrong BK, Baxter C, Duarte Romero B, Ebeling P, English DR, et al. The D-Health Trial: A randomized trial of vitamin D for prevention of mortality and cancer. Contemp Clin Trials. 2016;48:83-90. doi: 10.1016/j.cct.2016.04.005. PubMed PMID: 27086041.
20. Kawahara T, Suzuki G, Inazu T, Mizuno S, Kasagi F, Okada Y, et al. Rationale and design of Diabetes Prevention with active Vitamin D (DPVD): a randomised, double-blind, placebo-controlled study. BMJ Open. 2016;6(7):e011183. doi: 10.1136/bmjopen-2016-011183. PubMed PMID: 27388357; PubMed Central PMCID: PMCPMC4947789.
21. Ponticelli C, Sala G. Vitamin D: a new player in kidney transplantation? Expert Rev Clin Immunol. 2014;10(10):1375-83. doi: 10.1586/1744666X.2014.949674. PubMed PMID: 25135092.
22. Sarno G, Daniele G, Tirabassi G, Chavez AO, Ojo OO, Orio F, et al. The impact of vitamin D deficiency on patients undergoing kidney transplantation: focus on cardiovascular, metabolic, and endocrine outcomes. Endocrine. 2015;50(3):568-74. doi: 10.1007/s12020-015-0632-8. PubMed PMID: 25999028.
23. de Zeeuw D, Agarwal R, Amdahl M, Audhya P, Coyne D, Garimella T, et al. Selective vitamin D receptor activation with paricalcitol for reduction of albuminuria in patients with type 2 diabetes (VITAL study): a randomised controlled trial. Lancet. 2010;376(9752):1543-51. doi: 10.1016/S0140-6736(10)61032-X. PubMed PMID: 21055801.
24. Gonzalez E, Rojas-Rivera J, Polanco N, Morales E, Morales JM, Egido J, et al. Effects of oral paricalcitol on secondary hyperparathyroidism and proteinuria of kidney transplant patients. Transplantation. 2013;95(7):e49-52. PubMed PMID: 23545517.
25. Eleftheriadis T, Antoniadi G, Liakopoulos V, Kartsios C, Stefanidis I, Galaktidou G. Paricalcitol reduces basal and lipopolysaccharide-induced (LPS) TNF-alpha and IL-8 production by human peripheral blood mononuclear cells. Int Urol Nephrol. 2010;42(1):181-5. doi: 10.1007/s11255-009-9541-1. PubMed PMID: 19259778.
26. Park JW, Bae EH, Kim IJ, Ma SK, Choi C, Lee J, et al. Paricalcitol attenuates cyclosporine-induced kidney injury in rats. Kidney Int. 2010;77(12):1076-85. PubMed PMID: 20237458.
27. Donate-Correa J, Henriquez-Palop F, Martin-Nunez E, Hernandez-Carballo C, Ferri C, Perez-Delgado N, et al. Anti-inflammatory profile of paricalcitol in kidney transplant recipients. Nefrologia. 2017;37(6):622-9. doi: 10.1016/j.nefro.2017.03.028. PubMed PMID: 28623033.
28. Oblak M, Mlinsek G, Kandus A, Buturovic-Ponikvar J, Arnol M. Effects of paricalcitol on biomarkers of inflammation and fibrosis in kidney transplant recipients: results of a randomized controlled trial. Clin Nephrol. 2017;88(13):119-25. doi: 10.5414/CNP88FX26. PubMed PMID: 28664839.
29. Pihlstrom HK, Gatti F, Hammarstrom C, Eide IA, Kasprzycka M, Wang J, et al. Early introduction of oral paricalcitol in renal transplant recipients. An open-label randomized study. Transpl Int. 2017;30(8):827-40. doi: 10.1111/tri.12973. PubMed PMID: 28436117.

30. Batmanov K, Wang W, Bjoras M, Delabie J, Wang J. Integrative whole-genome sequence analysis reveals roles of regulatory mutations in BCL6 and BCL2 in follicular lymphoma. Sci Rep. 2017;7(1):7040. doi: 10.1038/s41598-017-07226-4. PubMed PMID: 28765546; PubMed Central PMCID: PMCPMC5539289.
31. Wang J. Computational biology of genome expression and regulation--a review of microarray bioinformatics. J Environ Pathol Toxicol Oncol. 2008;27(3):157-79. doi: 10.1615/jenvironpatholtoxicoloncol.v27.i3.10. PubMed PMID: 18652564.
32. Mathieu C, Adorini L. The coming of age of 1,25-dihydroxyvitamin D(3) analogs as immunomodulatory agents. Trends Mol Med. 2002;8(4):174-9. PubMed PMID: 11927275.
33. Veldman CM, Cantorna MT, DeLuca HF. Expression of 1,25-dihydroxyvitamin D(3) receptor in the immune system. Arch Biochem Biophys. 2000;374(2):334-8. doi: 10.1006/abbi.1999.1605. PubMed PMID: 10666315.
34. Nieuwland AJ, Kokje VB, Koning OH, Hamming JF, Szuhai K, Claas FH, et al. Activation of the vitamin D receptor selectively interferes with calcineurin-mediated inflammation: a clinical evaluation in the abdominal aortic aneurysm. Lab Invest. 2016;96(7):784-90. doi: 10.1038/labinvest.2016.55. PubMed PMID: 27239732.
35. Lucisano S, Arena A, Stassi G, Iannello D, Montalto G, Romeo A, et al. Role of Paricalcitol in Modulating the Immune Response in Patients with Renal Disease. Int J Endocrinol. 2015;2015:765364. doi: 10.1155/2015/765364. PubMed PMID: 26451144; PubMed Central PMCID: PMCPMC4586963.
36. Alborzi P, Patel NA, Peterson C, Bills JE, Bekele DM, Bunaye Z, et al. Paricalcitol reduces albuminuria and inflammation in chronic kidney disease: a randomized double-blind pilot trial. Hypertension. 2008;52(2):249-55. doi: 10.1161/HYPERTENSIONAHA.108.113159. PubMed PMID: 18606901.
37. Pinera-Haces C, Izquierdo-Ortiz MJ, Martin-de Francisco AL, Garcia-Unzueta MT, Lopez-Hoyos M, Toyos C, et al. Double treatment with paricalcitol-associated calcifediol and cardiovascular risk biomarkers in haemodialysis. Nefrologia. 2013;33(1):77-84. doi: 10.3265/Nefrologia.pre2012.Sep.11533. PubMed PMID: 23364629.
38. Navarro-Gonzalez JF, Donate-Correa J, Mendez ML, de Fuentes MM, Garcia-Perez J, Mora-Fernandez C. Anti-inflammatory profile of paricalcitol in hemodialysis patients: a prospective, open-label, pilot study. J Clin Pharmacol. 2013;53(4):421-6. doi: 10.1002/jcph.19. PubMed PMID: 23426718.
39. Mansouri L, Lundwall K, Moshfegh A, Jacobson SH, Lundahl J, Spaak J. Vitamin D receptor activation reduces inflammatory cytokines and plasma MicroRNAs in moderate chronic kidney disease - a randomized trial. BMC Nephrol. 2017;18(1):161. doi: 10.1186/s12882-017-0576-8. PubMed PMID: 28511692; PubMed Central PMCID: PMCPMC5434555.
40. Perez V, Sanchez-Escuredo A, Lauzurica R, Bayes B, Navarro-Munoz M, Pastor MC, et al. Magnetic bead-based proteomic technology to study paricalcitol effect in kidney transplant recipients. Eur J Pharmacol. 2013;709(1-3):72-9. doi: 10.1016/j.ejphar.2013.03.040. PubMed PMID: 23567070.
41. Tan X, Li Y, Liu Y. Paricalcitol attenuates renal interstitial fibrosis in obstructive nephropathy. J Am Soc Nephrol. 2006;17(12):3382-93. doi: 10.1681/ASN.2006050520. PubMed PMID: 17082242.
42. Amer H, Griffin MD, Stegall MD, Cosio FG, Park WD, Kremers WK, et al. Oral paricalcitol reduces the prevalence of posttransplant hyperparathyroidism: results of an open label randomized trial. Am J Transplant. 2013;13(6):1576-85. doi: 10.1111/ajt.12227. PubMed PMID: 23601186.
43. Swetha R, Gayen C, Kumar D, Singh TD, Modi G, Singh SK. Biomolecular basis of matrix metallo proteinase-9 activity. Future Med Chem. 2018;10(9):1093-112. doi: 10.4155/fmc-2017-0236. PubMed PMID: 29676173.
44. Jonsson S, Lundberg A, Kalvegren H, Bergstrom I, Szymanowski A, Jonasson L. Increased levels of leukocyte-derived MMP-9 in patients with stable angina pectoris. PLoS One. 2011;6(4):e19340. doi: 10.1371/journal.pone.0019340. PubMed PMID: 21559401; PubMed Central PMCID: PMCPMC3084822.

45. Feng X, Lv C, Wang F, Gan K, Zhang M, Tan W. Modulatory effect of 1,25-dihydroxyvitamin D 3 on IL1 beta -induced RANKL, OPG, TNF alpha , and IL-6 expression in human rheumatoid synoviocyte MH7A. Clin Dev Immunol. 2013;2013:160123. doi: 10.1155/2013/160123. PubMed PMID: 24348674; PubMed Central PMCID: PMCPMC3855937.
46. Hansen D, Rasmussen K, Rasmussen LM, Bruunsgaard H, Brandi L. The influence of vitamin D analogs on calcification modulators, N-terminal pro-B-type natriuretic peptide and inflammatory markers in hemodialysis patients: a randomized crossover study. BMC Nephrol. 2014;15:130. doi: 10.1186/1471-2369-15-130. PubMed PMID: 25112372; PubMed Central PMCID: PMCPMC4136403.
47. Boyce BF, Xing L. Biology of RANK, RANKL, and osteoprotegerin. Arthritis Res Ther. 2007;9 Suppl 1:S1. doi: 10.1186/ar2165. PubMed PMID: 17634140; PubMed Central PMCID: PMCPMC1924516.
48. Lee J, Lee S, Lee CY, Seo HH, Shin S, Choi JW, et al. Adipose-derived stem cell-released osteoprotegerin protects cardiomyocytes from reactive oxygen species-induced cell death. Stem Cell Res Ther. 2017;8(1):195. doi: 10.1186/s13287-017-0647-6. PubMed PMID: 28931423; PubMed Central PMCID: PMCPMC5606035.
49. Rochette L, Meloux A, Rigal E, Zeller M, Cottin Y, Vergely C. The Role of Osteoprotegerin and Its Ligands in Vascular Function. Int J Mol Sci. 2019;20(3). doi: 10.3390/ijms20030705. PubMed PMID: 30736365; PubMed Central PMCID: PMCPMC6387017.
50. Rochette L, Meloux A, Rigal E, Zeller M, Cottin Y, Vergely C. The role of osteoprotegerin in the crosstalk between vessels and bone: Its potential utility as a marker of cardiometabolic diseases. Pharmacol Ther. 2018;182:115-32. doi: 10.1016/j.pharmthera.2017.08.015. PubMed PMID: 28867452.
51. Toffoli B, Pickering RJ, Tsorotes D, Wang B, Bernardi S, Kantharidis P, et al. Osteoprotegerin promotes vascular fibrosis via a TGF-beta1 autocrine loop. Atherosclerosis. 2011;218(1):61-8. doi: 10.1016/j.atherosclerosis.2011.05.019. PubMed PMID: 21679949.
52. Mortlock S. Matrix metalloprotease-9: defining a normal reference range. Sch J App Med Sci. 2016;4:4146-50.
53. Dunkler D, Haller M, Oberbauer R, Heinze G. To test or to estimate? P-values versus effect sizes. Transpl Int. 2019. doi: 10.1111/tri.13535. PubMed PMID: 31560143.

Figure 1

Click here to access/download;Figure;Fig 1. Changes in levels of osteoprotegerin.pptx

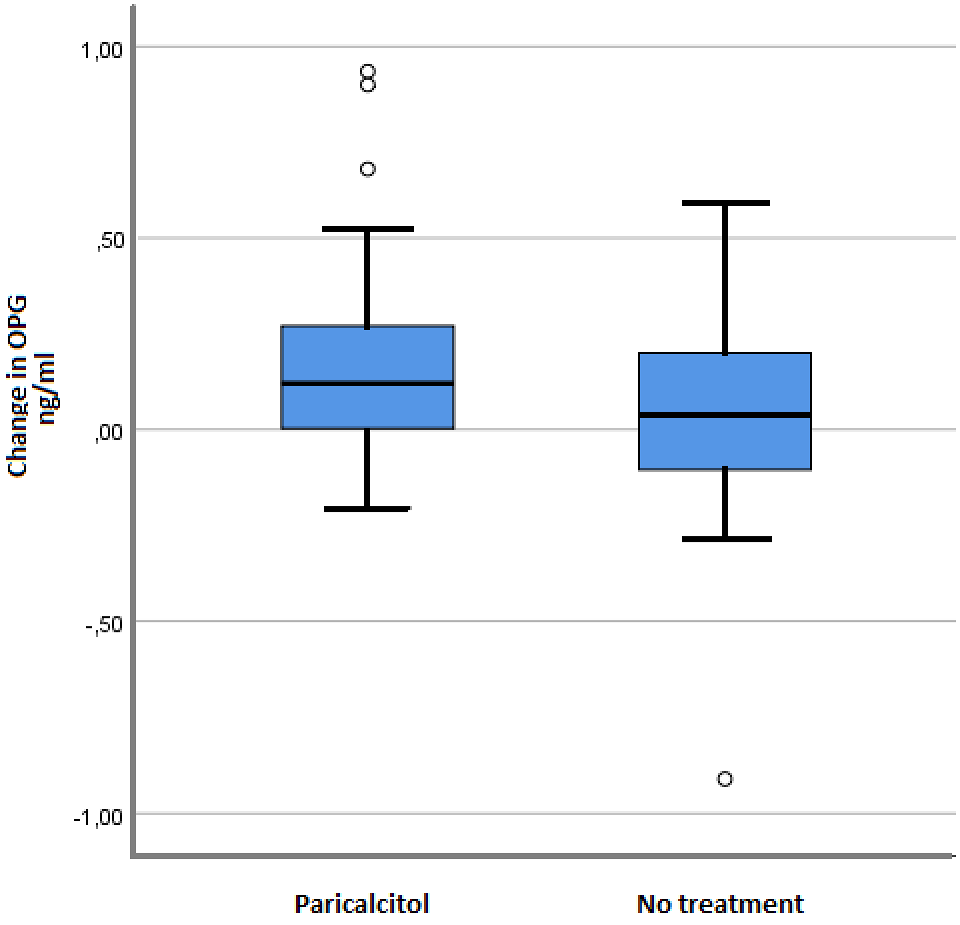

Figure 2

Click here to access/download;Figure;Fig.2 Heat map of gene expression levels.pptx

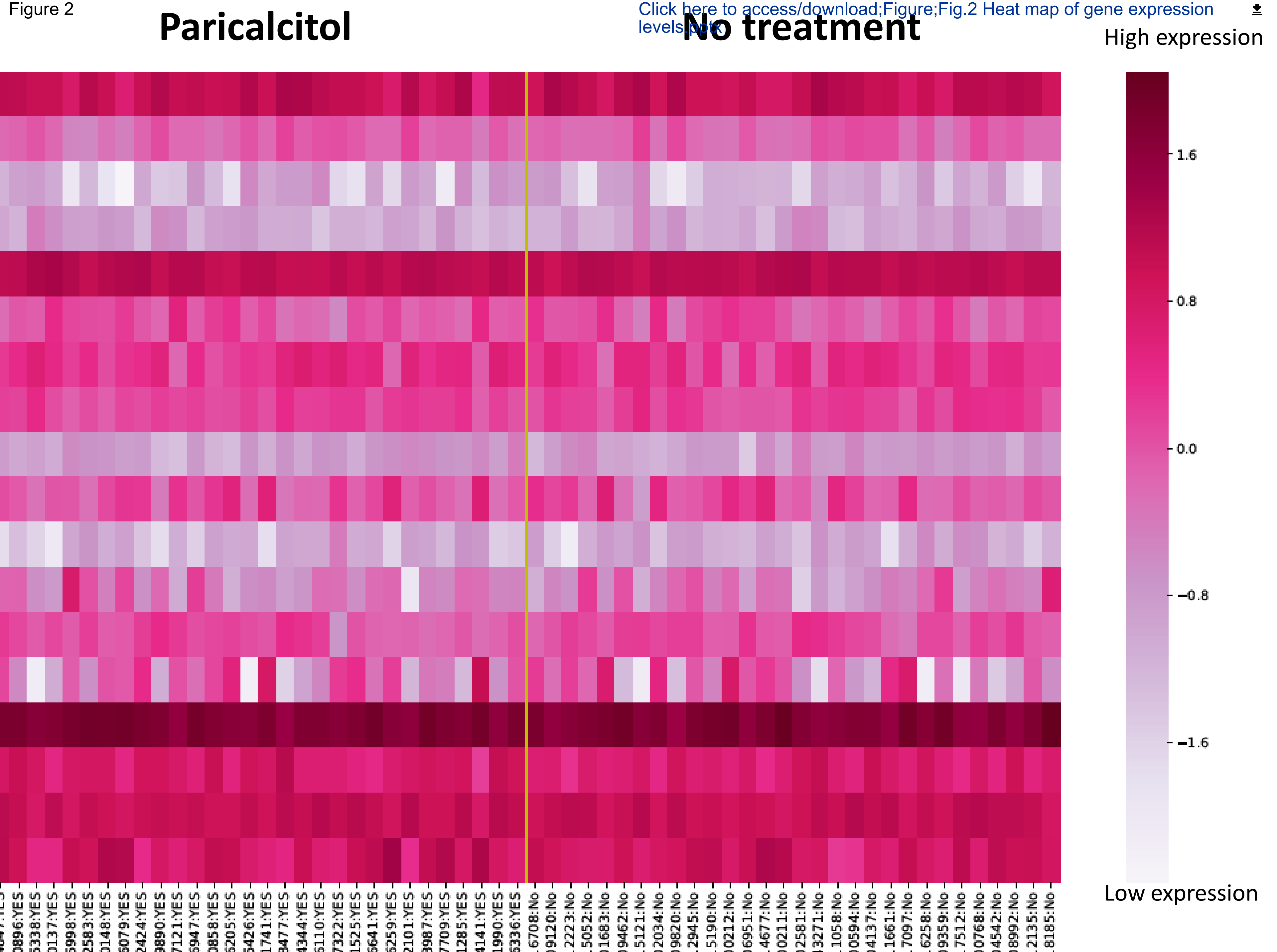

Project protocol (2013)

Click here to access/download
Supporting Information
Project protocol Paricalcitol.doc

CONSORT CHECKLIST for main trial_ref 30

Click here to access/download
Supporting Information
CONSORT Checklist 180716.doc

CONSORT diagram

Click here to access/download
Supporting Information
Consort diagram.bmp
